# Extracellular Vesicle Signatures and Post-Translational Protein Deimination in Purple Sea Urchin (*Strongylocentrotus purpuratus*) Coelomic Fluid—Novel Insights into Echinodermata Biology

**DOI:** 10.3390/biology10090866

**Published:** 2021-09-03

**Authors:** Stefania D’Alessio, Katherine M. Buckley, Igor Kraev, Polly Hayes, Sigrun Lange

**Affiliations:** 1 Tissue Architecture and Regeneration Research Group, School of Life Sciences, University of Westminster, London W1W 6UW, UK; w1650366@my.westminster.ac.uk (S.D.); p.hayes@westminster.ac.uk (P.H.); 2 Department of Biological Sciences, Auburn University, Auburn, AL 36849, USA; kbuckley@auburn.edu; 3 Electron Microscopy Suite, Faculty of Science, Technology, Engineering and Mathematics, Open University, Milton Keynes MK7 6AA, UK; igor.kraev@open.ac.uk; 4 UCL EGA Institute for Women’s Health, Maternal and Fetal Medicine, London WC1E 6AU, UK

**Keywords:** purple sea urchin (*Strongylocentrotus purpuratus*), protein deimination/citrullination, peptidylarginine deiminase (PAD), extracellular vesicles (EVs), coelomic fluid, immunity, metabolism, gene regulation

## Abstract

**Simple Summary:**

The purple sea urchin (*Strongylocentrotus purpuratus*) is a marine invertebrate that populates the east side of the Pacific Ocean from Mexico to Alaska, inhabiting intertidal and near-shore subtidal waters. Due to their ancient relationship with vertebrates, sea urchins are an important research model for developmental biology, cell biology, and immunology, as well as for understanding regenerative responses and ageing. This study assessed a specific protein modification called deimination/citrullination, which can alter protein function, allowing proteins to take on multiple and variable roles in different processes related to health and disease. This study also identified how extracellular vesicles, which are lipid blebs released from cells that participate in key processes for cell communication in health and disease, can carry proteins, including such modified protein cargo. This study may furthermore provide a platform for novel biomarker development to assess sea urchin health, which could be further applied, including for the monitoring of environmental changes.

**Abstract:**

The purple sea urchin (*Strongylocentrotus purpuratus*) is a marine invertebrate of the class Echinoidea that serves as an important research model for developmental biology, cell biology, and immunology, as well as for understanding regenerative responses and ageing. Peptidylarginine deiminases (PADs) are calcium-dependent enzymes that mediate post-translational protein deimination/citrullination. These alterations affect protein function and may also play roles in protein moonlighting. Extracellular vesicles (EVs) are membrane-bound vesicles that are released from cells as a means of cellular communication. Their cargo includes a range of protein and RNA molecules. EVs can be isolated from many body fluids and are therefore used as biomarkers in physiological and pathological responses. This study assessed EVs present in the coelomic fluid of the purple sea urchin (*Strongylocentrotus purpuratus*), and identified both total protein cargo as well as the deiminated protein cargo. Deiminated proteins in coelomic fluid EVs were compared with the total deiminated proteins identified in coelomic fluid to assess putative differences in deiminated protein targets. Functional protein network analysis for deiminated proteins revealed pathways for immune, metabolic, and gene regulatory functions within both total coelomic fluid and EVs. Key KEGG and GO pathways for total EV protein cargo furthermore showed some overlap with deimination-enriched pathways. The findings presented in this study add to current understanding of how post-translational deimination may shape immunity across the phylogeny tree, including possibly via PAD activity from microbiota symbionts. Furthermore, this study provides a platform for research on EVs as biomarkers in sea urchin models.

## 1. Introduction

The purple sea urchin (*Strongylocentrotus purpuratus*) is a marine invertebrate within the phylum Echinodermata. Together with the chordates and hemichordates (acorn worms), echinoderms (sea urchins, sea cucumbers, sea stars, brittle-stars) are part of the deuterostome superphylum, which originated 650–760 million years ago [1]. The common name, purple sea urchin, is given by its deep purple colour; this species populates the east side of the Pacific Ocean from Mexico to Alaska, inhabiting intertidal and near-shore subtidal waters [2,3]. Over the past century, sea urchins have served as an important research model in developmental biology, particularly the areas of regeneration and ageing. Echinoderms species show considerable regenerative abilities and significant variation in longevity (life spans range from 5–over 100 years, depending on species). Notably, however, both long- and short-lived sea urchins display negligible markers of cellular senescence and therefore do not fit within the classic understanding of biological ageing [3]. Analysis of the sea urchin genome [2] not only confirmed their relationship with vertebrates but also revealed great insights into immune function in this basal deuterostome [1,4,5]. The purple sea urchin immune system consists of two defence mechanisms that mirror those in vertebrates: a physical/chemical barrier; and a second barrier made of humoral factors, which triggers a humoral response and a consequent activation of phagocytic cells, antimicrobial factors, and inflammatory responses [6]. From a molecular standpoint, sea urchins have an ancient complement activation system that resembles the vertebrate complement systems [4,7,8,9], a significantly expanded array of pattern recognition receptors encoded in the genome, and a unique set of immune effector proteins [4,9,10,11]. This complex and sophisticated immune system may contribute to the ability of sea urchins to survive in hazardous conditions and adapt to different marine environments [12]. However, investigations into post-translational protein modifications are scarce. Roles for phosphorylation, glycosylation, and various other post-translational modifications have been reported [13,14,15,16], but hitherto no studies have assessed putative roles for post-translational deimination, which in chordates is caused by peptidylarginine deiminases (PADs) and/or by PAD homologues (arginine deiminases, ADI) in bacteria, protists, and fungi.

PADs are a phylogenetically conserved calcium-dependent family of enzymes responsible for citrullination/deimination, a post-translational modification that targets proteins in the cytoplasm, nucleus, and mitochondria, causing changes in the protein functions, structure and, consequently, in protein–protein interactions [17,18,19,20]. In mammals, five PADs isozymes have been identified (PAD1, 2, 3, 4 and 6), which display tissue-specific expression, with PAD2 being the most ubiquitously expressed. PAD2 is also considered the most phylogenetically conserved PAD. PAD2-like proteins have been identified in fish, as well as in amphibians, while PAD1, PAD2, and PAD3 are present in birds and reptiles [17,21,22,23,24,25]. In bacteria, parasites, and fungi, PAD homologues, also referred to as arginine deiminases (ADI), have been reported [26,27,28,29,30,31]. Many of the bacterial and parasitic PADs/ADI do show closest similarity to mammalian PAD6, followed by PAD2 [31]. Echinoderm PAD-like proteins have not been described, although a PAD/ADI protein has been reported in a marine bacterium (*Marixanthomonas ophiurae,* family *Flavobacteriaceae)* isolated from deep-sea brittle stars (Ophiuroidea) [32], as well as an ADI (AWW29909.1) in the bacterium *Echinicola strongylocentroti*, isolated from another sea urchin species, *S*. *intermedius*. Furthermore, ADI are reported in cyanobacteria, which form part of sea urchin, and other Echinodermata, diet. Proteins most susceptible to deimination include beta sheet structures and intrinsically disordered proteins, while the position of the arginine is also of some importance [33,34]. Target proteins for deimination include cytoskeletal, mitochondrial, and nuclear proteins and deimination can contribute to neo-epitope generation, triggering inflammatory responses including also the formation of neutrophil extracellular traps (NETosis) via histone H3 deimination, and can furthermore via histone deimination also affect gene regulation [18,35]. Protein deimination may also contribute to protein moonlighting, allowing the same protein to carry out different functions within a single polypeptide chain [36,37].

A recent comparative body of research has focused on identifying putative roles for PADs/ADI and downstream deimination of proteins involved in shaping immunity and metabolism in a wide range of taxa throughout the phylogenetic tree, including Alveolata [31], Mollusca [38], Crustacea [39], Merostomata [40], Agnatha [41], Chondrichthyes [24], Teleosts [23,42,43,44,45,46], Reptilia [25], Aves [47], Rodents [48,49], and sea and land mammals including Cetaceans [50], Pinnipeds [51], Artiodactyla [52,53], and Camelidae [54]. Through these studies, our group has particularly focused on identifying deimination signatures in circulatory fluid (e.g., plasma, serum, haemolymph or coelomic fluid, depending on species) and on characterising circulating extracellular vesicles (EVs), including with respect to their protein, including deiminated protein (and in some cases microRNA) cargos. EVs are membrane vesicles released from cells that play important roles in cellular communication in health and disease via the transfer of proteomic and genetic (including non-coding RNAs) cargo between cells [20,30,55,56]. EVs are found in most body fluids where they can readily be isolated and their cargo content can be useful biomarkers. Indeed, deiminated protein cargo in EVs may play functional roles in cellular communication and the regulation of shaping immunity and metabolic pathways, as well as gene regulation in a range of species. Therefore, an investigation into EV protein cargo as well as deimination signatures across phylogeny may be of considerable interest, including in the sea urchin.

In this aim, the current study isolated and characterised sea urchin coelomic fluid EVs, and assessed total proteomic and post-translationally deiminated protein cargo. Deimination signatures of the coelomic fluid were also assessed in comparison to the EV cargo. The reported findings provide novel insights into the post-translational regulation of pathways involved in immunity and metabolism of the purple sea urchin, as well as informing EV-mediated pathways in cellular communication. Our findings may further current understanding of the roles of post-translational modifications in the functional diversification of conserved proteins related to immunity, gene regulation, and metabolism throughout phylogeny.

## 2. Materials and Methods

### 2.1. Coelomic Fluid Sampling from Purple Sea Urchin

Coelomic fluid was isolated from three adult animals by inserting a chilled, 20-gauge syringe into the peristomial membrane. Whole coelomic fluid was mixed (1:1) with calcium–magnesium-free seawater (CMFSW; 460 mM NaCl, 10.73 mM KCl, 7.04 mM Na_2_SO_4_, 2.38 mM NaHCO_3_, pH = 7.4) containing 30 mM EDTA. To remove coelomocytes from the samples, whole coelomic fluid was centrifuged at 5000× *g* for 5 min. Cell-free coelomic fluid was collected, aliquoted, and frozen at −80 °C until use. All procedures were carried out according to protocols approved by the Auburn University Institutional Animal Care and Use Committee (2020).

### 2.2. Isolation of Extracellular Vesicles (EVs) and Nanoparticle Tracking Analysis (NTA)

Sea urchin coelomic fluid EVs were isolated from the coelomic fluid of three individual animals using sequential centrifugation and ultracentrifugation, according to previously described protocols [39] and following the Minimal Information for Studies of Extracellular Vesicles 2018 (MISEV2018) recommendations [57]. EVs were prepared from individual samples by diluting 200 µL of coelomic fluid in 300 µL of Dulbecco’s PBS (DPBS, ultrafiltered using a 0.22 µm filter, before use) and centrifuging for 20 min at 4000× *g* at 4 °C to remove aggregates and apoptotic bodies. The supernatants were then collected and ultra-centrifuged for 1 h at 100,000× *g* at 4 °C to obtain the EV-enriched pellets; thereafter, each pellet was resuspended (“washed”) in 500 µL DPBS and ultra-centrifuged again at 100,000× *g* at 4 °C for 1 h. Finally, the resulting EV pellets were resuspended in 100 µL of DPBS and 10 µL used for nanoparticle tracking analysis (NTA), and added to 990 µL DPBS to measure EV profiles at a dilution of 1:100. To generate EV size distribution profiles and to quantify EVs, the NanoSight NS300 system (Malvern Panalytical Ltd., Malvern, UK) was used. Syringe speed 50 was applied; four repetitive reads of 60 s were recorded for each sample with the camera settings at level 13 for recoding, while the post-analysis threshold setting was set at 5. Replicate histograms were generated from the videos, presenting mean and confidence intervals of the four recordings for each sample, using the NanoSight software 3.0 (Malvern).

### 2.3. Transmission Electron Microscopy (TEM)

Sea urchin coelomic fluid EVs were also assessed by TEM, similar as to previously described methods [39]. Thawed EV pellets were resuspended in 100 mM sodium cacodylate buffer (pH 7.4) and one drop (~3–5 μL) was placed onto a grid with a previously glow discharged carbon support film. The EV suspension was partially air-dried (~10 min) and thereafter the sample was fixed for 1 min at room temperature, placing the grid onto a drop of a fixative solution (2.5% glutaraldehyde in 100 mM sodium cacodylate buffer; pH 7.0). The grid was then applied to the surface of three drops of distilled water to wash the EV sample; excess water was removed using filter paper. Staining of the EVs was carried out for 1 min using 2% aqueous Uranyl Acetate (Sigma-Aldrich, Gillingham, UK); excess stain was removed using filter paper and air drying the grid. EVs were imaged by TEM using a JEOL JEM 1400 transmission electron microscope (JEOL, Tokyo, Japan), which was operated at 80 kV, using a magnification of 30,000× to 60,000×. Recording of digital images was carried out using an AMT XR60 CCD camera (Deben, Bury Saint Edmunds, UK).

### 2.4. Isolation of Deiminated Proteins Using F95-Enrichment

Isolation of total deiminated proteins from sea urchin coelomic fluid and EVs was carried out using the F95 pan-deimination antibody (MABN328, Merck, Feltham UK), which detects citrullinated proteins and has been developed against a deca-citrullinated peptide [58], in conjunction with the Catch and Release^®^ v2.0 immunoprecipitation kit (Merck), according to previously described methods [47]. Coelomic fluid from three individual animals (3 × 100 µL) and the corresponding EV isolates (3 × 50 µL) from the same animals were used for F95 enrichment, which was performed on a rotating platform at 4 °C overnight. The deiminated (F95-bound) proteins were eluted under reducing conditions using the elution buffer provided with the Catch and Release^®^ v2.0 immunoprecipitation kit, according to the manufacturer’s instruction (Merck). The eluted protein fractions were collected and diluted 1:1 in 2× Laemmli sample buffer (BioRad, Watford, UK). The samples were stored at −20 °C until used for SDS-PAGE analysis, in-gel digestion for LC-MS/MS analysis, and Western blotting, as described in Section 2.5, Section 2.6 and Section 2.7.

### 2.5. Western Blotting Analysis

SDS-PAGE was carried out on the sea urchin coelomic fluid as well as on the EVs. All samples were diluted 1:1 in a denaturing 2× Laemmli sample buffer (containing 5% beta-marcaptoethanol, BioRad, UK) and then boiled for 5 min at 100 °C. Protein separation was carried out on 4–20% gradient TGX gels (BioRad, UK) at 165 V for 50 min. Western blotting was performed for 1 h at 15 V using a Trans-Blot^®^ SD semi-dry transfer cell (BioRad, UK). Following blotting, the nitrocellulose membranes were stained with PonceauS (Sigma, UK) to ensure even protein transfer. Blocking was performed at room temperature for 1 h with 5% bovine serum albumin (BSA, Sigma, UK) in tris buffered saline (TBS) containing 0.1% Tween20 (BioRad, UK) (TBS-T). For the detection of a putative PAD/ADI-like protein in purple sea urchin coelomic fluid, the anti-human PAD2 antibody (ab50257, Abcam, Cambridge, UK; diluted 1/1000 in TBS-T) was applied. Furthermore, antibodies against the other four human PAD isozymes were also tested for cross-reaction with sea urchin coelomic fluid (PAD1 (ab181762, 1/1000 in TBS-T), PAD3 (ab50246, 1/1000 in TBS-T), PAD4 (ab50247, 1/1000 in TBS-T), and PAD6 (PA5–72059, Thermo Fisher Scientific, Hemel Hempstead, UK, 1/1000 in TBS-T; results are shown in Supplementary Figure S2). For characterisation of coelomic fluid EVs, two phylogenetically conserved EV markers were used: CD63 (ab68418; diluted 1/1000) and Flotillin-1 (ab41927; diluted 1/2000), which have, besides in human, been previously shown to cross-react with EVs from other taxa [24,25,39,40,41,48]. Primary antibody incubation was carried out on a shaking platform overnight at 4 °C. The nitrocellulose membranes were thereafter washed at room temperature in TBS-T for 3 × 10 min and then incubated with HRP-conjugated secondary anti-rabbit IgG antibody (BioRad), diluted 1/3000 in TBS-T, for 1 h at room temperature. Subsequent washing of membranes was performed at room temperature for 4 × 10 min in TBS-T, followed by a final wash in TBS without Tween20. Protein bands were digitally visualised using enhanced chemiluminescence (ECL, Amersham, UK) and the UVP BioDoc-ITTM System (Thermo Fisher Scientific, Dartford, UK).

### 2.6. Silver Staining

SDS-PAGE (using 4–20% gradient TGX gels, BioRad, UK) was carried out under reducing conditions for the F95-enriched protein eluates from both coelomic fluid and EVs, as well as for whole protein cargo from EVs, and whole protein of the coelomic fluid. The gels were silver stained following SDS-PAGE using the BioRad Silver Stain plus Kit (1610449, BioRad, UK), performed according to the manufacturer’s instructions.

### 2.7. Liquid Chromatography with Tandem Mass Spectrometry (LC-MS/MS) Analysis of EV Protein Cargo and Deiminated Protein Hits in Sea Urchin Coelomic Fluid and EVs

Liquid chromatography with tandem mass spectrometry (LC-MS/MS) was carried out to identify total protein content from coelomic fluid EVs and to identify F95-enriched proteins from both coelomic fluid and EVs (using a pool of coelomic fluid or EVs from *n* = 3 individual animals, respectively), according to previously described methods [25,40,54]. The F95-enriched protein preparations as well as total protein from EVs were diluted 1:1 in 2× Laemmli buffer and boiled for 5 min at 100 °C, and run 0.5 cm into a 12% TGX gel (BioRad, UK), excising the concentrated protein bands (containing the whole F95 eluate from coelomic fluid and the coelomic fluid EVs respectively, as well as total EV protein) using a scalpel. The gel bands were trypsin digested and subjected to proteomic analysis using a Dionex Ultimate 3000 RSLC nanoUPLC (Thermo Fisher Scientific Inc., Waltham, MA, USA) system in conjunction with a QExactive Orbitrap mass spectrometer (Thermo Fisher Scientific Inc., Waltham, MA, USA). The LC-MS/MS analysis was performed by Cambridge Proteomics (Cambridge, UK), using previously described procedures [25,40,54]. Post-run, the data were processed using Protein Discoverer (version 2.1., Thermo Scientific) and the MS/MS data were converted to mgf files, which were submitted to the Mascot search algorithm (Matrix Science, London, UK) to identify protein hits. Search for protein hits was conducted against the UniProt database for purple sea urchin (CCP_Strongylocentrotus_purpuratus Strongylocentrotus_purpuratus_20210510; 34,423 sequences; 23,911,872 residues) and against a common Echinidea UniProt database CCP_Echinidea Echinidea_20210511 (38,194 sequences; 24,939,030 residues). An additional search was conducted against a common contaminant database (cRAP 20,190,401; 125 sequences; 41,129 residues). The fragment and peptide mass tolerances were set to 0.1 Da and 20 ppm, respectively, and the threshold value for significance was set at *p* < 0.05. The peptide cut-off score applied was 33, to indicate identity or extensive similarity. Datasets were submitted to PRIDE, according to standards by MIAPE (https://www.psidev.info/miape accessed on 25 July 2021).

### 2.8. Protein–Protein Interaction Network Analysis

To predict putative protein–protein interaction networks associated with the deiminated protein hits from purple sea urchin coelomic fluid and EVs, STRING analysis (Searching Tool of Retrieval of Interacting Genes/Proteins; https://string-db.org/ accessed on 25 July 2021) was performed. A similar analysis was also performed for total protein cargo of the EVs. Protein networks were generated based on protein names and applying the function of “searching multiple proteins” in STRING using the Echinoidea protein database available in STRING, with *Strongylocentrotus purpuratus* as the representative species. STRING analysis parameters were set at “medium confidence” and “basic settings”. The colour lines connecting the nodes for the network edges represent the following evidence-based interactions: “known interactions” (these are based on experimentally determined curated databases) and “predicted interactions” (these are based on gene co-occurrence, gene neighbourhood, via text-mining, gene fusion, protein homology, or co-expression). Kyoto Encyclopedia of Genes and Genomes (KEGG) and Gene Ontology (GO) pathways for the predicted protein networks (both for F95-enriched proteins in EVs and coelomic fluid, as well as for total protein cargo in EVs) were then also assessed in STRING. Pathways were colour coded using the analysis tool (see corresponding colour code key included for individual nodes and connective lines for each network analysis figure). Analysis of predicted disordered regions was furthermore carried out for the deiminated protein hits using the FoldIndex© analysis tool (https://fold.weizmann.ac.il/fldbin/findex accessed on 25 July 2021) [59].

### 2.9. Statistical Analysis

The NTA curves were generated using the Nanosight 3.0 software (Malvern, UK), showing mean (black line) and standard error of mean (SEM); confidence intervals are indicated by a red line. Protein–protein interaction networks were constructed using the STRING (https://string-db.org/ accessed on 25 July 2021) analysis tool, applying basic settings and medium confidence. Significance was considered as *p* < 0.05.

### 2.10. Echinoderm Genome and Transcriptome Data Mining for PAD Orthologs

Searches for PAD orthologs were conducted using BLAST searches for *S. purpuratus* and other echinoderms on NCBI (https://www.ncbi.nlm.nih.gov/ accessed on 25 July 2021) and Echinobase (https://www.echinobase.org accessed on 25 July 2021). Following this, the *S. purpuratus* genome and the other available echinoderm genome assemblies were further interrogated for PAD/PAD-like genes based on conservation in PAD protein domains using tBLASTn searches (https://blast.ncbi.nlm.nih.gov/Blast.cgi?PROGRAM=tblastn accessed on 25 July 2021) [22] with human PAD protein sequences (PAD1–4, PAD6). Any resultant scaffold matches were then used to predict PAD/PAD-like sequences using the FGENESH gene finder tool in Softberry (http://www.softberry.com accessed on 25 July 2021) and predicted PAD/PAD-like protein sequences were checked using BLASTp searches for accuracy of identification. Available Echinodermata transcriptional data (e.g., expressed sequence tag and transcriptome shotgun assembly datasets) were also checked using the same approach to detect any potential expression of PAD/PAD-like protein genes to account for any potential missing data in any of the current echinoderm genome assemblies.

## 3. Results

### 3.1. Characterisation of Sea Urchin Coelomic Fluid EVs

**Figure 1:**
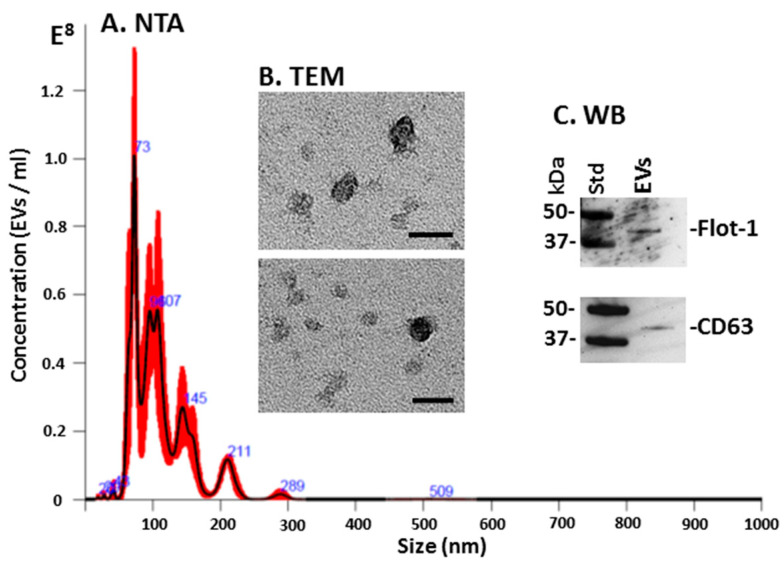
*Strongylocentrotus purpuratus* coelomic fluid EV profile. (**A**) Nanoparticle tracking analysis (NTA) showing a representative size distribution profile of sea urchin coelomic fluid EVs, with the majority of EVs within a 30–300 nm range. (**B**) Transmission electron microscopy (TEM) from sea urchin coelomic fluid EVs; scale bar represents 50 nm. (**C**) Western blotting analysis (WB) showing sea urchin coelomic fluid EVs positive for Flot-1 and CD63, respectively; the molecular weight for the standard is indicated in kilodaltons (kDa).

The particle number and size distribution of sea urchin coelomic fluid EVs were assessed using the NanoSight NS300. The analysis showed a poly-dispersed EV population in the size range of 30–300 nm with the majority of EVs present in the size range of 50–150 nm (Figure 1A). Coelomic fluid EV yield between the three samples showed some individual variability in the range of 1.53 × 10^9^–3.33 × 10^10^ particles/mL. EVs were further assessed by transmission electron microscopy (TEM; Figure 1B) and Western blotting showed positive bands for the EV-specific markers CD63 and Flot-1 (Figure 1C), therefore meeting the minimum criteria for the characterisation of EVs as per specifications by the International Society for Extracellular Vesicles [57].

### 3.2. LC-MS/MS Analysis of Total EV Cargo from Purple Sea Urchin

Total protein cargo from sea urchin coelomic fluid EVs was analysed by LC-MS/MS. Protein hits identified against the Echinoidea UniProt database are presented in Table 1. Furthermore, detailed LC-MS/MS results (also including results from a common contaminant database; cRAP 20,190,401; 125 sequences; 41,129 residues) are provided in Supplementary Table S1.

### 3.3. Protein–Protein Interaction Networks for Total EV Protein Cargo from Purple Sea Urchin

**Figure 2:**
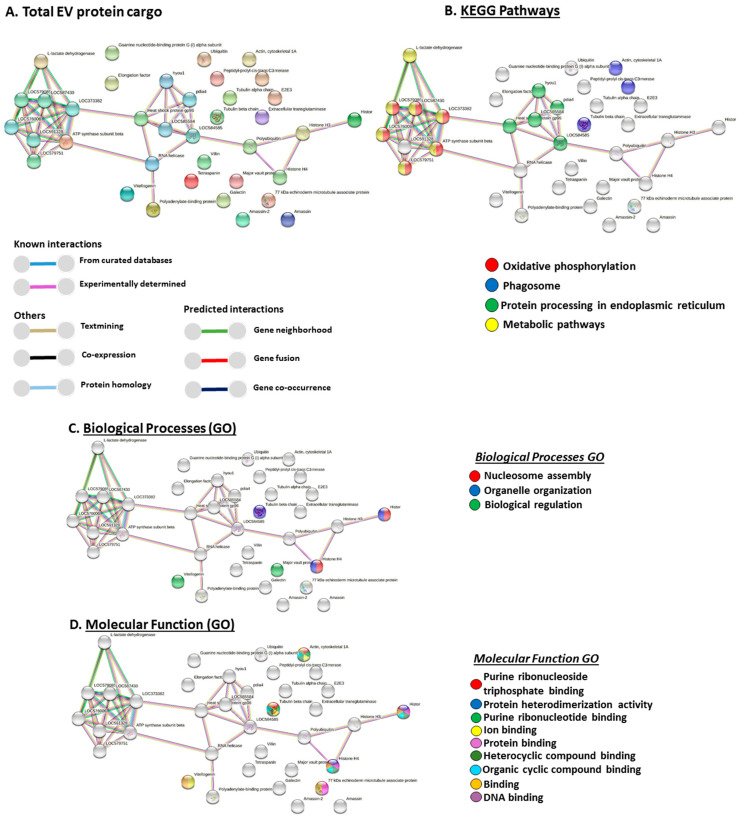
Protein–protein interaction networks of total EV protein cargo identified in purple sea urchin. (**A**) Functional protein networks are based on known and predicted interactions in Echinoidea using STRING analysis. Coloured nodes represent query proteins only. Coloured lines connecting nodes show the type of interactions between the nodes of the networks based on known interactions, predicted interactions, and others (including text-mining, co-expression, and protein homologue); colour code legend is provided in the figure. PPI enrichment *p*-value for the protein network is 9.25 × 10^−5^. (**B**) KEGG pathways identified from STRING analysis for EV total protein cargo (annotated hits). (**C**) Gene Ontology (GO) Biological processes identified from STRING analysis for total EV protein cargo (annotated hits). (**D**) GO Molecular Function pathways identified from STRING analysis for total EV protein cargo (annotated hits; protein names of hits are presented in the figures; additional interacting proteins are: LOC579085 = ATP synthase subunit gamma, mitochondrial; LOC587430 = ATP synthase subunit O, mitochondrial; LOC373382 = ATP synthase subunit alpha; LOC576006 = ATP synthase subunit delta, mitochondrial; LOC579751 = ATP synthase F(0) complex subunit B1, mitochondrial).

The total protein cargo from EVs was assessed using STRING analysis to identify KEGG and GO pathways relating to EV-mediated cellular communication. For the prediction of protein–protein interaction networks, protein names were submitted to STRING (Search Tool for the Retrieval of Interacting Genes/Proteins) analysis (https://string-db.org/ accessed on 25 July 2021). Protein interaction networks were built based on known and predicted interactions and represent all proteins identified in *Strongylocentrotus purpuratus* EVs, based on proteins from the STRING protein database for Echinoidea. The following networks were identified as represented in Figure 2.

### 3.4. PAD Protein Homologue and Deiminated Proteins in Purple Sea Urchin Coelomic Fluid and EVs

**Figure 3:**
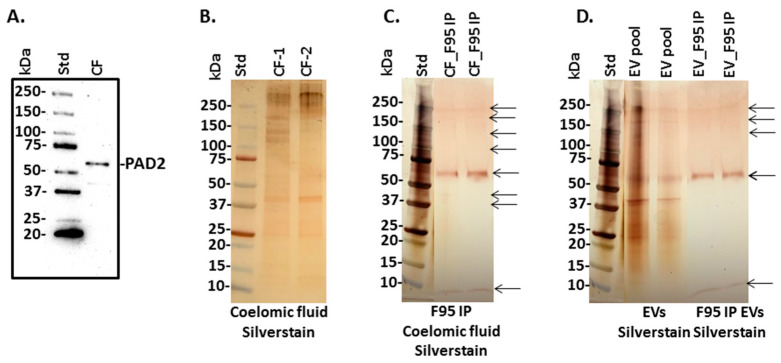
Peptidylarginine deiminase (PAD)-like protein detection and deiminated proteins in sea urchin coelomic fluid. (**A**) Using the human PAD2-specific antibody, a strong positive band was detected in coelomic fluid (CF). (**B**,**C**) Coelomic fluid (CF) was silver-stained for total protein (**B**); the F95-enriched proteins from coelomic fluid (CF) are shown in (**C**)—protein bands for F95-enriched proteins are highlighted with arrows. (**D**) Showing total proteins as detected by silver staining in EVs isolated from sea urchin coelomic fluid (a pool of 3 samples was used for the EV enriched fraction) and citrullinated/deiminated proteins were isolated from the EVs using F95 enrichment (EV_F95 IP); arrows point at main F95-enriched protein bands. The molecular weight standard (Std) is shown on the left hand side of each blot/gel and indicated in kilodaltons (kDa).

For the detection of a putative PAD homologue from sea urchins, anti-human PAD2-specific antibody was used in Western blotting to assess any cross-reaction with a sea urchin PAD-like protein, as PAD2 is considered the most phylogenetically conserved PAD isozyme. A positive protein band was detected at approximately 65–70 kDa in coelomic fluid (Figure 3A), while the expected PAD protein size in mammals is around 70 kDa. Proteins of total coelomic fluid were stained by silver staining, following SDS-PAGE (Figure 3B), and F95-enriched fractions from total coelomic fluid were also analysed by SDS-PAGE and silver staining, showing bands at sizes ranging from 10–250 kDa (Figure 3C). Silver staining of total protein content from coelomic fluid EVs (Figure 3D “EV-pool”) showed more protein abundance than the F95-enriched fractions from the EVs, which revealed bands at 10 kDa alongside several bands in the range of 60–250 kDa (Figure 3D).

### 3.5. LC-MS/MS Analysis of Deiminated Proteins in Purple Sea Urchin Coelomic Fluid and EVs

Deiminated protein identification in sea urchin coelomic fluid and EVs (see Figure 3C,D) was carried out following F95 enrichment using LC-MS/MS analysis. Deiminated protein hits identified with Echinoidea are summarised in Table 2 for EVs and CF; further detailed analysis on numbers of matches and total scores for F95-enriched proteins from EVs and CF are provided in Supplementary Tables S2 and S3, respectively. In addition, full LC-MS/MS results (also including data from a common contaminant database; cRAP 20,190,401; 125 sequences; 41,129 residues) are reported in Supplementary Tables S4 and S5, respectively.

### 3.6. Protein–Protein Interaction Network Identification of Deiminated Proteins in Purple Sea Urchin Coelomic Fluid and EVs

**Figure 4:**
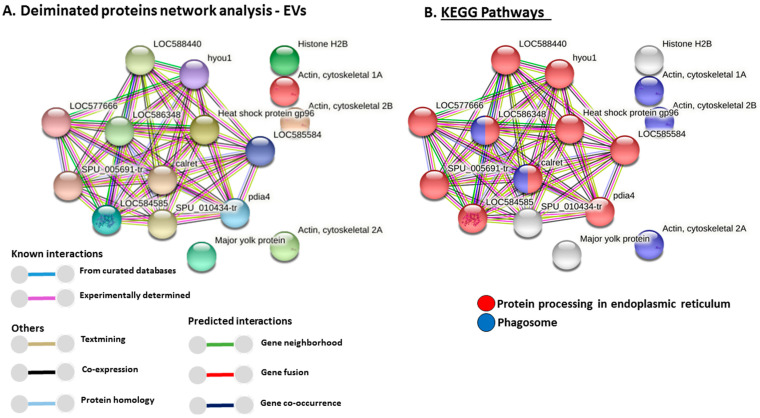

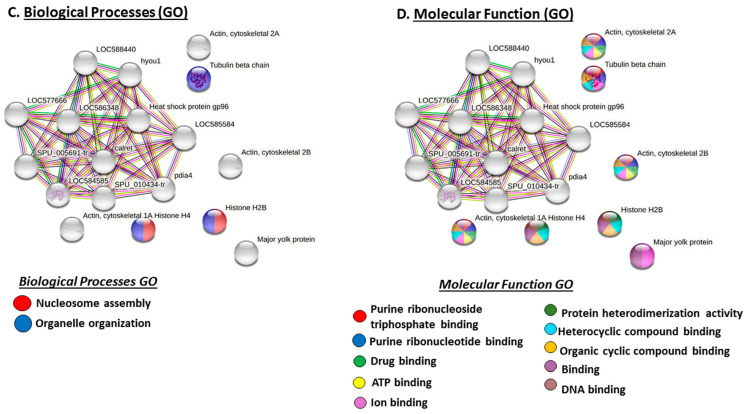
Protein–protein interaction networks of deiminated proteins identified in purple sea urchin coelomic fluid EVs. (**A**) Protein networks for deiminated proteins in purple sea urchin EVs based on known and predicted interactions in Echinoidea, using STRING analysis. Coloured nodes represent query proteins only. Coloured lines connecting nodes show the type of interactions between the nodes of the networks based on known interactions, predicted interactions, and others (including text-mining, co-expression, and protein homologue); colour code legend is provided in the figure. PPI enrichment *p*-value for the protein network is 1.11 × 10^−16^. (**B**) KEGG pathways identified from STRING analysis for the deiminated protein candidates in purple sea urchin EVs. (**C**) Gene Ontology (GO) Biological processes identified from STRING analysis for deiminated protein candidates in purple sea urchin EVs. (**D**) GO Molecular functions identified from STRING analysis for deiminated proteins candidates in purple sea urchin EVs. Protein names of hits listed in the tables are presented in the figures; additional interacting proteins are: LOC588440 = dnaJ homolog subfamily B member 11; LOC577666 = dnaJ homolog subfamily C member 1; LOC584585 = endoplasmic reticulum chaperone BiP; hyou1 = heat shock protein 70 family member; LOC586348 = uncharacterised protein(Calnexin-like); SPU_005691-tr = Protein disulfide-isomerase; SPU_010434-tr = annotation not available (part of calriticulin protein network); pdia4 = Protein disulfide-isomerase A4.

**Figure 5:**
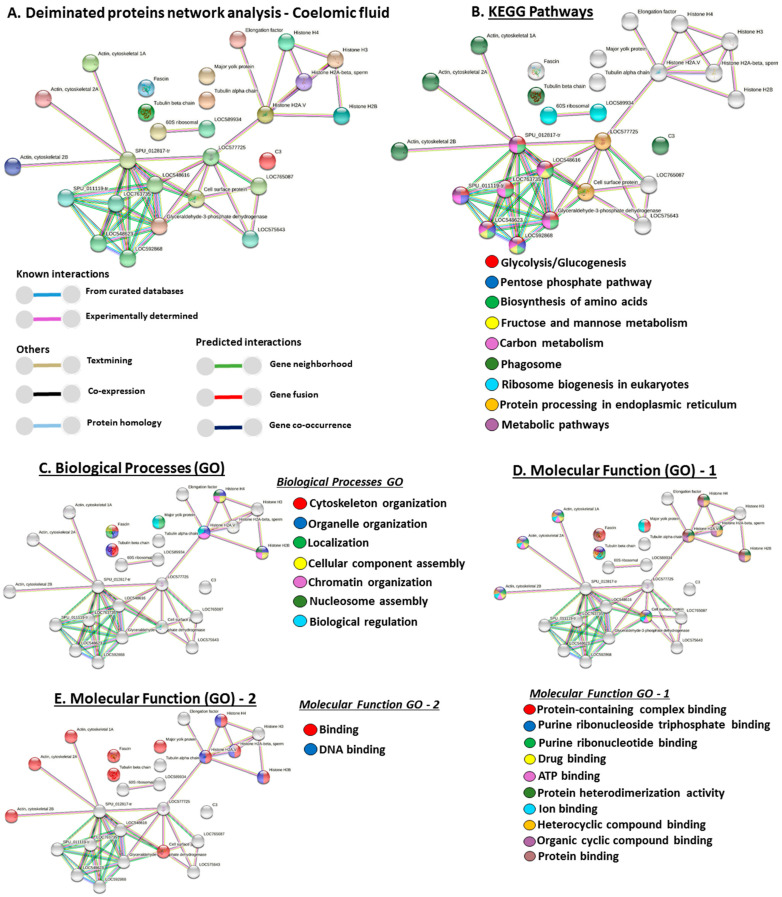
Protein–protein interaction networks of deiminated proteins identified in purple sea urchin coelomic fluid. (**A**) Protein networks for deiminated proteins in purple sea urchin coelomic fluid based on known and predicted interactions in Echinoidea, using STRING analysis. Coloured nodes represent query proteins only. Coloured lines connecting nodes show the type of interactions between the nodes of the networks based on known interactions, predicted interactions, and others (including text-mining, co-expression, and protein homologue); colour code legend is provided in the figure. PPI enrichment *p*-value for the protein network is 5.31 × 10^−5^. (**B**) KEGG pathways identified from STRING analysis for the deiminated protein candidates in purple sea urchin coelomic fluid. (**C**) Gene Ontology (GO) Biological processes identified from STRING analysis for deiminated protein candidates in purple sea urchin coelomic fluid. (**D**,**E**) GO Molecular functions identified from STRING analysis for deiminated proteins candidates in purple sea urchin coelomic fluid. Protein names of hits listed in the tables are presented in the figures; additional interacting proteins are: LOC589934 = large subunit GTPase 1 homolog; LOC577725 = heat shock protein 83; LOC765087 = Hsp70/Hsp90-organising protein; LOC575643 = activator of heat shock 90kDa protein ATPase homolog 1; LOC763735 = Pyruvate kinase; LOC548623 = Fructose-bisphosphate aldolase; LOC592868 = Fructose-bisphosphate aldolase, non-muscle type like; SPU_012817-tr = Phosphoglycerate kinase; SPU_011119-tr = Glucose-6-phosphate isomerase.

For the prediction of protein–protein interaction networks of deiminated proteins identified in purple sea urchin coelomic fluid and EVs, protein names were submitted to STRING (Search Tool for the Retrieval of Interacting Genes/Proteins) analysis (https://string-db.org/ accessed on 25 July 2021). Protein interaction networks were built based on known and predicted interactions and represent all deiminated proteins identified in *Strongylocentrotus purpuratus* coelomic fluid and EVs, respectively. The networks were based on proteins from the STRING protein database for Echinoidea. Protein interaction networks are presented below for deiminated proteins from EVs (Figure 4) and whole coelomic fluid (Figure 5).

**Figure 6:**
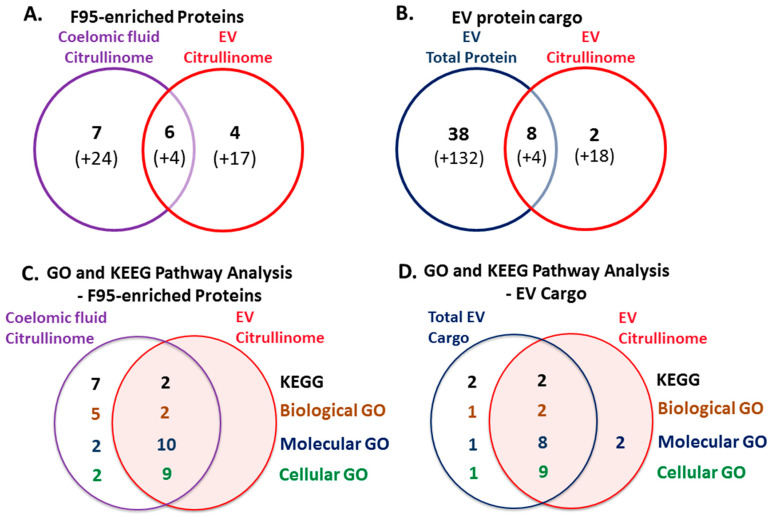
Deiminated protein hits and EV cargo and associated STRING protein network analysis of purple sea urchin coelomic fluid citrullinome and EV citrullinome, and of total EV protein cargo. (**A**) Venn diagram showing deiminated protein hits (“citrullinome”) identified in *Strongylocentrotus purpuratus* coelomic fluid and EVs, representing shared and unique proteins (uncharacterised proteins are indicated in brackets), identified using the Echinoidea UniProt database. (**B**) Venn diagram showing protein hits identified for total EV proteome cargo and deiminated EV cargo (EV citrullinome) protein hits. (**C**) Venn diagram showing KEGG and GO pathway analysis for deiminated proteins identified in coelomic fluid (coelomic fluid citrullinome) and EVs (EV citrullinome), respectively. (**D**) Venn diagram showing GO and KEGG pathways for total EV protein cargo and shared pathways with the EV citrullinome. The number of specific and overlapping pathways is indicated and relates to the STRING networks in Figure 2, Figure 4 and Figure 5.

Venn diagrams summarising common and specific deimination hits and associated protein networks identified for deiminated protein hits in coelomic fluid and EVs, as well as EV total protein cargo, are shown in Figure 6.

### 3.7. FoldIndex© Analysis of Deiminated Protein Hits in Sea Urchin Coelomic Fluid and EVs

As disordered proteins have been reported to be more susceptible to deimination [33,34], deiminated protein hits identified for purple sea urchin coelomic fluid and EVs were further assessed for disordered regions using the FoldIndex© analysis tool (https://fold.weizmann.ac.il/fldbin/findex accessed on 25 July 2021) [59]. A summary for predicted disordered regions, which were identified in the protein hits, as well as the number of arginines present in the corresponding sea urchin protein sequences identified here, is summarised in Table 3 and Table 4 for deiminated protein hits identified in EVs and coelomic fluid, respectively.

### 3.8. PADs Identified from Mining Echinoderm Genomes

General BLAST searches for PAD orthologs against *S. purpuratus* and other members of the Echinodermata revealed no hits. The tBLASTn searches of the *S. purpuratus* genome data also resulted in no hits for PAD/PAD-like genes. The same searches against all other echinoderm genome assemblies also resulted in no hits apart from one scaffold match for the mottled brittle starfish *Ophionereis fasciata* (GCA_900067615). A BLASTp search with the FGENESH predicted protein from *O. fasciata* revealed identity matches with putative PADs in Cyanobacteria (Table 5). No evidence of PAD/PAD-like gene expression was found from searches of available echinoderm transcriptional datasets.

## 4. Discussion

The current study is the first to characterise extracellular vesicles (EVs) and associated proteomic cargo of sea urchin coelomic fluid and to assess the presence of protein deimination signatures in sea urchin coelomic fluid and EVs.

EVs isolated from purple sea urchin coelomic fluid were found to be poly-dispersed in the size range of mainly 30–300 nm, with the majority of EVs falling in the size range of 50–150 nm, which is indicative of a high proportion of small EVs, or “exosomes”, while EV peaks at larger size (up to 300 nm) were also observed (medium/large EVs, “microvesicles”). The sea urchin EVs showed positive for the phylogenetically conserved EV-specific markers CD63 and Flot-1, and were further verified by transmission electron microscopy (TEM). The EV characterisation therefore meets the minimum requirements for EV characterisation by the International Society for Extracellular Vesicle research [57].

For the assessment of a putative PAD/ADI-like protein in sea urchin coelomic fluid, the anti-human PAD2 specific antibody was used and revealed the presence of an approximately 65–70 kDa band, while mammalian PAD2 would be expected at approximately 70–75 kDa. Reports of Echinoidea PAD-like proteins are scarce, and while searching the Echinoid database no reported PAD-like protein hit was present in the protein database. Therefore, the current study provides the first report of a PAD/ADI-like protein in sea urchin coelomic fluid, via cross-reaction with human PAD antibodies. However, genome and transcriptome mining results in this study indicate that there are no PAD/PAD-like protein coding genes represented in sea urchin genomes, nor across the Echinodermata. PAD/ADI-like proteins have though been reported in Echinoidea microbiota [32]. In the current study, the only PAD coding gene identified from an echinoderm genome assembly was identified as a Cyanobacteria PAD. It is highly accepted that genome assemblies for most organisms will contain genomic contamination as a result of the unintended sequencing of symbionts, parasites, and food sources [60]. This has been shown particularly in other invertebrates, including arthropods where sequences of fungi, protists, and bacteria had originally been incorporated into the genome assembly and initially interpreted as being arthropod derived [60].

The presence of deiminated protein products in coelomic fluid was assessed by F95 enrichment, using the pan-citrulline/deimination F95 antibody [58] and protein hits were identified by LC-MS/MS analysis. Some deiminated target proteins overlapped between whole coelomic fluid and EVs, while others were specific for coelomic fluid or EVs, respectively. A total of 41 deiminated proteins (including non-annotated hits) were identified in coelomic fluid of purple sea urchin, whereof six were overlapping with deiminated protein hits: two annotated target proteins (Major yolk protein, and 60S ribosomal protein L40) and four uncharacterised proteins with a secondary annotation (Cytoskeletal Actin-1A, -2A, -1B, and -2B; Histone H2B; Histone H4 and Tubulin beta chain). Seven annotated deiminated target proteins were identified to be unique for coelomic fluid (Complement C3; Late histone H2B.2.1; Tubulin alpha chain, Fascin; Elongation factor alpha-1; Glyceraldehyde-3-phosphate dehydrogenase and Cell surface protein). Furthermore, 31 (including non-annotated hits) deiminated protein hits were identified in EVs, whereof four deiminated target proteins were identified as unique for EVs (Beta actin, Cytoplasmic actin CyII, gp96 heat shock protein, and 98K protein).

Protein interaction networks for deiminated proteins revealed nine KEGG pathways relating to immune (phagosome) and metabolic (glycolysis/gluconeogenesis, pentose phosphate pathway, biosynthesis of amino acids, fructose and mannose metabolism, carbon metabolism, ribosome biogenesis, protein processing in ER, metabolic pathways) functions in coelomic fluid, and two of these KEGG pathways were also found in the EVs and related to immune function (phagosome) and metabolic (protein processing) function. Compared with KEGG pathways identified for deiminated proteins in other species, the glycolysis/gluconeogenesis pathway was previously identified in cetacean sera [50], in plasma-EVs from naked mole-rat [48], in alligator plasma-EVs [25], in lamprey plasma and plasma-EVs [41], and in lobster and horseshoe crab haemolymph [39,40], as well as in alveolates [31]. The KEGG pentose phosphate pathway was previously identified for deiminated proteins from cetacean sera [50] and in alveolates [31]. The biosynthesis of amino acids KEGG pathway was previously identified for deiminated proteins from bovine plasma and plasma-EVs [52], plasma and plasma-EVs of reindeer [53], naked mole-rats [48], and lampreys [41], from cetacean sera [50], and lobster haemolymph [39]. The fructose and mannose metabolism KEGG pathway was identified for deiminated proteins in cetacean sera [50], lamprey plasma [41], and in alveolates [31]. The carbon metabolism KEGG pathway has been identified for deiminated proteins in lamprey plasma [41], in lobster and horseshoe crab haemolymph [39,40], as well as in alveolates [31]. The ribosome KEGG pathway was previously related to F95-enriched proteins from alligator plasma-EVs [25], and lamprey plasma and plasma-EVs [41]. The protein processing in ER pathway was previously also identified for deiminated proteins in lamprey plasma-EVs [41]. The phagosome KEGG pathway was previously identified for deiminated proteins in reindeer plasma [53], bovine serum and serum-EVs [52], cetacean sera [50], and in lobster haemolymph [39].

Further GO pathways for F95-enriched proteins included cytoskeletal, nuclear, and metabolic function, with seven biological GO pathways, whereof five were specific for coelomic fluid (cytoskeleton organisation, localisation, cellular component assembly, chromatin organisation, biological regulation) and a further two were shared with EVs (nucleosome assembly, organelle organisation). Deiminated protein networks revealed two molecular GO pathways specific to coelomic fluid (protein-containing complex binding and protein binding), while a further 10 molecular GO pathways were shared with EVs (purine ribonucleoside triphosphate binding, purine ribonucleotide binding, drug binding, ATP binding, ion binding, protein heterodimerisation activity, heterocyclic compound binding, organic cyclic compound binding, binding, DNA binding). Cellular component GO pathways for deimination-enriched proteins (Supplementary Figure S1A,B) showed two specific pathways for coelomic fluid (nuclear chromatin, polymeric cytoskeletal fibre), and a further nine pathways shared with EVs (cytoskeleton, cytoplasm, intracellular non-membrane-bound organelle, intracellular organelle, cell, nucleosome, protein-containing complex, intracellular membrane-bound organelle, nucleus).

In addition to F95-enriched (deiminated) proteins, LC-MS/MS analysis was carried for the total protein cargo of sea urchin EVs; here, a total of 182 protein hits were identified, whereof eight overlapped with deiminated hits from EVs (Major yolk protein, Beta actin, Actin, Tubulin beta chain, Histones H2B and H4, Heat shock protein gp96, and 60S ribosomal protein L40). Furthermore, total EV cargo showed that some proteins that were found as deiminated in coelomic fluid (Complement C3, Actin cytoskeletal 1A-1B, Tubulin alpha chain, Tubulin beta chain, Histone H4, Histone H2B and Glyceraldehyde-3-phosphate dehydrogenase) are amongst the total protein cargo in EVs. This indicates differences in deimination targets between EVs and coelomic fluid and furthermore shows that a large number of proteins relating to many key cellular functions are exported as EV cargo; only some of those are deiminated in EVs.

Total protein EV cargo-related KEGG pathways had two overlapping pathways with deiminated EV cargo (phagosome, protein processing in ER) and two further KEGG pathways for total EV cargo only (oxidative phosphorylation and metabolic pathways).

Biological GO pathways for total EV protein cargo showed two overlapping GO pathways with deiminated EV protein cargo (nucleosome assembly, organelle organisation) and one additional GO pathway (biological regulation).

Molecular function GO pathways for total EV protein cargo were nine in total, whereof one was specific (protein binding) while eight overlapped with deimination-enriched pathways in EVs (purine ribonucleoside triphosphate binding, purine ribonucleotide binding, protein heterodimerization activity, ion binding, heterocyclic compound binding, organic cyclic compound binding, binding, DNA binding). Furthermore, two molecular GO pathways found in the F95-enriched EV protein networks (ATP binding and drug binding) were not enriched for total EV cargo.

Cellular component GO pathways for total EV protein cargo (Supplementary Figure S1C) were 10 in total, whereof one was specific to total protein cargo (cytoskeleton), while nine overlapped with deimination-enriched pathways in EVs (cytoplasm, cytoskeleton, intracellular organelle, intracellular non-membrane-bound organelle, intracellular membrane-bound organelle, cell, nucleus, nucleosome, and protein-containing complex).

These differences in EV total protein cargo versus the EV citrullinome indicate that the associated KEGG and GO pathways differ in sea urchin coelomic fluid. It must though be noted that due to a lack of annotation of a high number of protein hits both for the F95-enriched (deiminated) proteins as well as total protein cargo analysis, the current analysis is limited to the annotated hits only and may underestimate the number of pathways regulated both by EV communication as well as post-translational deimination in coelomic fluid and EVs.

Below, annotated target proteins of deimination identified in purple sea urchin in the current study are discussed in relation to their known functions in both sea urchins and the wider literature, where appropriate, to try to further understand putatively relevant roles for deimination on protein function in cellular communication, including throughout phylogeny.

**Major yolk protein** (**MYP**) was a common deiminated target protein in both coelomic fluid and EVs of purple sea urchin. MYP is one of the most abundant proteins in sea urchin eggs and yolk platelets, is present in coelomic fluid of both males and females, and its mainly synthesised in the intestine of adult sea urchins [61]. It has been established that MYP is a transferrin-like, iron binding protein [62] and also plays important roles in gametogenesis [63]. MYP has also been identified to have antimicrobial properties in sea urchins (*Lytechinus variegatus*), acting as part of the innate immune defence [64]. Furthermore, MYP plays roles in immune regulation by activating the TLR pathway in sea cucumbers (*Apostichopus japonicus*) [65]. MYP has not been reported as a deimination target before; it contains 15 predicted disordered regions and 63 arginines, which are potential targets of deimination. How it may be regulated via deimination for various functions in development and immunity remains to be investigated.

**60S ribosomal protein L40** was a deiminated protein hit in coelomic fluid and EVs (the hit was with *Psammechinus miliaris*). This protein component of the 60S subunit of the ribosome is encoded by the ubiquitin gene and is essential for the translation of a subset of cellular transcripts. As ribosomal proteins are structural components of the protein synthetic machinery, they play multifaceted and important roles in protein synthesis [66,67]. They have furthermore been related to innate, including mucosal, immune responses and can act as anti-microbials [68,69,70,71]; in sea urchins, they are for example linked to heat stress responses [72]. The heterogeneity of ribosomes has been highlighted to contribute to various roles in stem cells and development, including via rRNA modifications and post-translational modifications [73]. Ribosomes have previously been identified as deimination candidates in other taxa, including in humans [74], teleosts [22], agnathans [41], and mollusks [38]. Roles for deimination in the regulation of ribosomes may be of considerable interest across phylogeny, both in a physiological and pathological context.

Deiminated forms of several actins were identified in coelomic fluid and EVs. Actin is a key cytoskeletal cellular protein, with actin filaments playing important roles in secretory vesicle transport, in mitochondria, and in endosomes [75]. In sea urchins, actins have been implicated in diverse functions including embryogenesis [76], fertilisation [77], and cytoskeletal organisation in immune recognition processes [78]. **Actin cytoskeletal 1A**, **2A**, **1B**, and **2B** were identified as deiminated target proteins in both coelomic fluid and EVs. Assessing these targets, they all showed three predicted disordered regions, with a longest region of 17 aa, and all contained 18 arginines, which are potential targets for deimination. **Cytoplasmic Actin CyII** was deiminated in EVs (the hit was with *Heliocidaris erythrogramma*). This 361 aa protein contains 18 arginines, which could potentially be deiminated, and three disordered regions, with the longest region at 53 aa. Beta actin was identified as deiminated in EVs, although the protein hit was against *Mesocentrotus nudus*, where it contains three predicted disordered regions, a total of 37 disordered residues, whereof the longest disordered region is 17 aa. Arginine content is 18 arginines within the 376 aa protein. Actins have previously been identified as deimination candidates in other taxa, including Crustacea [39] and Mollusca [38], and actin deimination has in mammalian cells been associated with EV biogenesis [79]. Deimination may contribute to actins’ multifaceted functions in a range of physiological and pathological processes across phylogeny.

Several deiminated histones were identified in purple sea urchin in the present study. **Histones H2A**, **H2B**, **H3**, and **H4** were all identified to be deiminated in either coelomic fluid or EVs, or in both. These histones are reported in sea urchin embryos, larva, and adults [80], although deimination has not been assessed, but they are known deimination targets in other species, with roles in epigenetic regulation and anti-pathogenic responses in a range of taxa [25,48], as well as in gene regulation relating to various human pathologies, including cancers [20,81,82]. In sea urchins, histones are widely studied in development, where histone modifications such as phosphorylation and ubiquitination have also received considerable attention [14,83], while deimination has not been reported before in sea urchin histone research. Post-translational modifications of core histones, including methylation, phosphorylation, acetylation, and citrullination/deimination, may affect many of their functions in relation to chromatin structure, including effects on histone–histone and histone–DNA interactions, and may also affect chaperone binding [84].

**Histone H2A** was a deiminated target protein in sea urchin coelomic fluid. It has two predicted disordered regions, with the longest disordered region being 35 aa, while 12 arginines are found in the 125 aa protein. As other histones, H2A is a known deimination candidate. **Histone H2B** was a deiminated target protein in both coelomic fluid and EVs, while late histone HB2.2.1 was found in coelomic fluid. H2B has one disordered region of 51 residues and 8 out of 122 aa are arginines, making it quite a susceptible protein for deimination. Indeed, histones are well known deimination candidates. **Histone H3** was a deiminated target in coelomic fluid. It has one predicted disordered region of 66 residues, and the 136aa protein contains 18 arginines, which can act as candidates for deimination. **Histone H4** was a deiminated target protein in both coelomic fluid and EVs. It has one predicted disordered region of 44 residues, with 14 arginines out of 103 residues, and is therefore a strong deimination candidate. Histone H3 deimination is in many species associated with the trapping of foreign pathogens via extracellular traps, as reported in a range of taxa [85]. Histones can furthermore serve as antimicrobial compounds as reported in humans [86] and various other taxa including molluscs [87,88,89,90], crustaceans [91,92], amphibians [93], teleosts [94], reptiles [95], and pinnipeds [96]. However, histone H3 deimination has also been shown to relate to the loss of bactericidal activity [97]. Histone deimination is also a well-known factor in gene regulation, including in pluripotency [98], in development [99], and in various pathologies including cancers [100,101]. Histone H3 deimination is also related to neural regeneration [102,103] and neurodegenerative disease [49]. In sea urchins, histones (H1 and H2A) have been studied in relation to development and embryogenesis [14] and histone H3 post-translational phosphorylation in relation to development [104], but deimination has not previously been reported. The regulation of histones’ multifaceted functions, including by post-translational modifications such as deimination, requires further investigation throughout phylogeny. These may play roles in anti-pathogenic responses, as well as in gene regulation, tissue regeneration, and development.

**Heat shock protein gp96** (also known as glucose-regulated protein 96) was identified as a deimination hit in sea urchin EVs. This highly conserved ER-resident protein is part of the HSP90 family [105]. HSP90 family proteins have wide-ranging cellular functions including regulating the protein folding response, cell cycle control, and the regulation of stress-induced cell damage (including thermal stress and infection) as well as organismal development [106,107,108]. In sea urchins, heat shock protein expression is affected by stress-induced responses, including chronic heat stress [72,109]. The sea urchin Gp96 contains nine predicted disordered regions, whereof the longest region is 111 aa and contains 30 arginines (out of 806 residues), which are potential targets of deimination. In various animal and cellular models, Gp96 upregulation is observed in response to stressful stimuli, including glucose starvation, and in the ER it is a molecular chaperone for correcting unfolded proteins, is involved in the degradation of misfolded proteins, and participates in the activation of protein translation [110]. It furthermore has roles in antigen presentation and pro-inflammatory cytokine secretion, acting as a danger signal in innate and adaptive immunity. GP96 has roles in Ca^2+^ homeostasis and in insulin signalling pathways, and therefore with putative roles in cell growth and differentiation as well as ER stress responses. Interestingly, Gp96 is implicated in self-tolerance, and its upregulation has been linked to autoimmunity [110]. Roles for Gp96 in pro- and anti-tumour responses have been identified, and it has, for example, been found to promote glioma oncogenesis and progression, while tumour-derived Gp96 has been assessed as a candidate for tumour vaccination [105]. In the liver, Gp96 maintains liver development and hepatocyte function, while its pharmacological inhibition in vitro contributes to pro-oncogenic responses [111]. Such multifaceted roles for Gp96, both in physiological and pathological functions, could possibly be aided by post-translational changes, including deimination, and indeed this protein contains a high number of arginines, which can allow for deimination-mediated structural and functional changes. While Gp96 has not been reported as a deimination candidate before to our knowledge, HSP has previously been reported as a deimination candidate in rheumatoid arthritis, facilitating deimination-induced shifts in protein structure that aid B cell tolerance bypassing [112]. In other taxa, HSP90 was identified as deiminated in camelid serum under normal physiological conditions [54], as well as in Mollusca haemolymph [38]. It may therefore be of considerable interest how this protein family may be regulated by deimination throughout phylogeny and how deimination may contribute to protein moonlighting functions.

Tubulins were identified as deiminated target proteins in sea urchins. **Tubulin beta chain** was a deiminated target protein in both coelomic fluid and EVs. It contains four predicted disordered regions, whereof the longest is 57 aa, and out of 447 residues, 20 arginines are found, which can be targets of deimination. **Tubulin alpha chain** was deiminated in coelomic fluid. It was found to have two predicted disordered regions, whereof the longest was 49aa, and moreover 20 arginines are present in the 452 aa protein. Tubulin plays roles in cytoskeletal rearrangement and has been studied in sea urchins, for example, in relation to cytoskeletal organisation in immune recognition [78], as well as in development and metamorphosis [113], where post-translational modifications such as acetylation, detyrosination, and polyglutamylation have been studied and found to contribute to the diversification of tubulin functions [114,115]. Sea urchins are furthermore a good model to study anti-proliferative drugs, including effects on tubulin dynamics [116]. The post-translational deimination of tubulin has hitherto not been reported in sea urchin, while tubulin has previously been identified as a deimination candidate in other species, including in llama [54] and lamprey [41], as well as being associated with EV biogenesis and release in mammalian cells, including via deimination [79]. Roles for PADs and pharmacological PAD inhibition have furthermore been linked to the regulation of EV biogenesis both in mammalian cells as well as in bacteria and parasites, indicative of a phylogenetically conserved function of deimination-mediated pathways in EV release [28,30,79,117,118,119,120]. The deimination of tubulin may contribute to EV-mediated processes for cellular communication across taxa, including in immune responses and in relation to various pathologies as well as in homeostasis. Exact roles for deimination in contributing to tubulin dynamics in sea urchin will require further investigation.

**Complement C3** was identified as deiminated in coelomic fluid. Notably, while C3 was a deimination candidate in coelomic fluid only, C3 also formed part of the EV cargo, albeit not in deiminated form. C3 is a key component of the complement system and has been well-described in sea urchins [4,6,7,8,9]. Sea urchin C3 was found to contain 15 predicted disordered regions, with a total of 258 disordered residues, the longest of which is 39 aa. Furthermore, 85 arginines, potentially targets of deimination, are found in the 1699 aa protein sequence. This highlights that C3 is very likely to undergo deimination and C3 deimination was recently reported for the first time in teleost fish [23]; it has since been identified in a range of taxa including elasmobranchs [24], reptiles [25], birds [47], cetaceans [50], pinnipeds [51], and artiodactyls [52,53]. Furthermore, in teleost fish, C3 has been found in deiminated form in both serum as well as in serum EVs [44,46] and in mucosal EVs [42], while in the current study C3 was a deimination candidate in coelomic fluid only, but not in the EVs, while C3 was identified as part of the total proteomic cargo in the EVs. This indicates that C3 may play differing roles in cellular communication in deiminated form, and also that export of its unmodified versus deiminated form in EVs may differ between animal species.

**Fascin** was identified as a deiminated target protein in coelomic fluid. It contains six predicted disordered regions, whereof the longest is 51 residues and the 496aa protein sequence contains 17 arginines, which can potentially be deiminated. It is a monomeric actin filament bundling protein, originally identified in sea urchin [121], and is found in three forms in vertebrates, with roles in regulating cytoskeletal structures for the maintenance of cell adhesion and motility [122]. It relates to developmental morphogenesis [123], has roles in nucleolar architecture [124], but is also linked to cell invasion in pathologies, including cancers [125,126], where it influences the formation of invadopodia and cancer metastasis [127]. Fascin is also essential for immunological synapse formation and is related to T regulatory cell adhesion to antigen presenting dendritic cells [128]. Fascin is furthermore linked to promoting wound healing via cell migration [129] and is down-regulated in neurological disease [130,131], while in neurodevelopment it contributes to promoting neuron formation and migration [132]. Fascin has been identified to be regulated by various post-translational modifications, mainly phosphorylation and ubiquitination [123], while the deimination of fascin has not been reported before but may contribute to hitherto unknown functions in multifaceted functions of fascin in health and disease across phylogeny.

Deiminated **elongation factor alpha-1** was detected in coelomic fluid. It contains two predicted disordered regions, whereof the longest is 27 residues. The 461 aa protein sequence contains 19 arginines. It has multiple roles in metabolic functions, including cell growth, cytoskeleton organisation, apoptosis, nuclear export of proteins, and the immune response [133,134,135,136]. In sea urchins, elongation factor 1 alpha has been found to undergo transcriptional and translational modifications in early developmental processes [137]. Elongation factor 1 alpha has also been identified as a biomarker for hypoxic stress, which can be problematic for several marine species, including due to eutrophication [138]. Previously, elongation factor alpha-1 has been identified as a deimination candidate in teleosts [22], Crustacea [39], and Mollusca [38]. The roles for deimination in multifaceted functions of elongation factor 1 alpha will need further exploration across taxa.

**Glyceraldehyde-3-phosphate dehydrogenase** (**GAPDH**) was a deiminated target protein in coelomic fluid. While no disordered regions were predicted, the 337 aa sequence contains 11 arginines, which may be candidates for deimination. GAPDH is an evolutionarily conserved enzyme [139] with key functions in the glycolytic pathway, and also with roles in nuclear RNA export, membrane fusion, and DNA repair [140,141]. It has previously been identified as deiminated in teleost fish [22], in Mollusca [38], and in Crustacea [39], as well as in relation to cancer [118]. The deimination of GAPDH may contribute to its multifaceted functions in health and disease in a range of species; to what extent deimination affects GAPDH function in sea urchins remains to be investigated.

Further deiminated protein hits were **cell surface protein**, which was found deiminated in coelomic fluid. The hit was against *Paracentrotus lividus*, and the protein contains five predicted disordered regions and 107 disordered residues, whereof the longest disordered region is 41 residues. The number of arginines is 34 out of the 370 aa protein. In sea urchins, it is linked to fertilisation and egg protein synthesis [142]. Furthermore, **98K protein** was identified as deiminated in EVs, and was a hit with *Hemicentrotus pulcherrimus*, but no specific functions have yet been reported for this protein in sea urchins.

The regulation of immune and metabolic proteins, as well as histones, via post-translational or epigenetic changes is of considerable interest, and previous studies in sea urchins have, for example, identified roles for cytidine deaminases, which modify cytidine and cause mutations in DNA by changing cytosine into uracil, leading to modulated immune responses of hosts and pathogens [143]. Furthermore, a range of post-translational modifications including acetylation, ubiquitination, and phosphorylation have been assessed in relation to various protein functions in sea urchins, as highlighted in the discussion above. However, deimination has hitherto not been reported in sea urchins.

While the current study assessed deimination signatures in the coelomic fluid of purple sea urchin, a PAD protein homologue was not found in the reported protein databases for sea urchins, or in the Echinoderm protein database. Additionally, upon the interrogation of available echinoderm genome assemblies and available transcriptomic data, no evidence of PAD/PAD-like protein coding genes in sea urchins or other echinoderms was found. Furthermore, the phylogenetic distribution of the PAD domain appears to be restricted to chordates, fungi, and bacteria [144]. This could be indicative of a lack of PAD orthologs in deuterostomes. However, further work beyond the scope of this is needed for verification. In the current study, the only PAD coding gene identified from an echinoderm genome assembly was identified as a Cyanobacteria PAD. Interestingly, PAD/ADI proteins have also been reported in microbiota of Echinoidea, including, for example, the marine bacterium *Marixanthomonas ophiurae,* family *Flavobacteriaceae* isolated from deep-sea brittle stars [32], and from *Echinicola strongylocentroti*, a bacterium isolated from sea urchin (*Strongylocentrotus intermedius*). Furthermore, ADI is well known in cyanobacteria [27], (also termed blue-green algae) which do contribute to sea urchin diet. At this stage, it cannot be excluded that the deiminated protein products observed here may be generated by ADI activity from microbiota in the coelomic fluid, including cyanobacteria. Indeed, in humans, bacteria have been shown to modulate some immune responses in the host, such as complement C5a [26], and possibly there may be a co-operation or symbiosis between commensals and/or pathogens and the host in the utilisation of ADI, as previously speculated also for Alveolata [31]. As the Echinoderm database did not reveal protein hits with human PAD protein sequences, but there are PAD-like (ADI) sequences reported from bacteria of echinoderms, including cyanobacteria, this may be of some interest. These bacterial ADI furthermore share similarity with human PAD6 and PAD2, and interestingly sea urchin coelomic fluid also showed cross-reactivity with human PAD6 antibodies (but less with the other PAD isozyme antibodies), as shown in Supplementary Figure S2, in addition to the cross-reaction with anti-human PAD2 as shown in Figure 2. Complex symbiotic associations between Echinodermata and the microbiota of their coelomic fluid, including the bioactivity of microbiota in sea urchin coelomic fluid, have in other studies pointed to roles in development, immunity, and metabolism [145,146,147,148,149]. Therefore, the possibility that deiminated protein products identified in this current study in coelomic fluid may most likely relate to host microbiota PAD/ADI activity cannot be excluded owing to the absence of functional PAD genes across the Echinodermata. This is of considerable interest and warrants further exploration.

Recent studies indicate the emergence of PADs within the chordates via horizontal gene transfer from cyanobacteria [150]. The lack of PAD orthologs in Echinoderms indicates that the transfer occurred after the major radiation events within the Deuterostoma and could explain the uniqueness of PADs in the chordate lineage. Furthermore, it has been shown that cyanobacteria PAD can actively citrullinate/deiminate mammalian proteins in a calcium-dependent manner [150], and this is in a similar vein as our suggestion here, based on the findings of our current study, that sea urchin proteins may be deiminated by cyanobacteria, or possibly other microbiota, PAD/ADI. The modulation of sea urchin immunity, metabolism, and gene regulation via deimination by PAD/ADI of microbiota could point to novel mechanisms in the regulation of key pathways in echinoderm biology.

As echinoderms are subjected to considerable environmental challenges, including due to pollutants and temperature changes, it will be interesting to investigate whether deimination signatures and EV profiles can act as indicative biomarkers to assess such stressors. It must be noted that the current study used a pool of three individual samples to establish a baseline for the identification of deiminated proteins in sea urchins, and therefore individual variation, including in response to stressors, will also need to be further assessed in future studies. Interestingly, in studies using teleost fish models to assess environmental temperature effects on immune responses [43], it has been found that EV and deimination signatures are modified in response to changed water temperatures. EV and/or deimination signatures may therefore hold potential as indicative markers for sea urchin health, including due to environmental effects.

## 5. Conclusions

The current study is the first to show deiminated protein signatures in Echinodermata, using the purple sea urchin as a model species. The findings indicate that numerous key immune, metabolic, and gene regulatory pathways are influenced by this post-translational modification and may contribute to their diverse functions. Furthermore, extracellular vesicles (EVs) from coelomic fluid were purified and characterised and their protein cargo analysed with respect to whole, as well as deiminated, protein cargo. This highlights roles for EVs in cellular communication for a range of immune and metabolic pathways via EV-mediated protein transport. EV and deimination signatures may possibly be developed as biomarkers in sea urchins, and furthermore inform the evolution of the PAD/ADI pathway in the phylogeny tree.

## Figures and Tables

**Table 1:** Total protein cargo of coelomic fluid EVs from purple sea urchin (*Strongylocentrotus purpuratus*), identified by liquid chromatography with tandem mass spectrometry (LC-MS/MS) analysis. Uncharacterised hits with a secondary hit that was annotated are included and indicated in brackets; other unidentified protein hits are not included in this table but can be found in Supplementary Table S1, alongside detailed LC-MS/MS results (also including data from a common contaminant database; cRAP 20,190,401; 125 sequences; 41,129 residues). Protein ID, protein name, species hit with the Echinoidea UniProt database, number of matches, and total score are included in the table. The analysis is based on a pool of 3 individual samples.

Protein ID	*Species Name*	Matches	Total Score
Protein Name	Common Name	(Sequences)	(*p* < 0.05) †
**P19615/MYP_STRPU**	*Strongylocentrotus purpuratus*	76	2443
Major yolk protein	Purple sea urchin	(75)	
**O44344_STRPU**	*Strongylocentrotus purpuratus*	65	2119
Complement component C3	Purple sea urchin	(53)	
**Q7Z1Y6_HEMPU**	*Hemicentrotus pulcherrimus*	54	1729
Major yolk protein	Sea urchin	(42)	
**Q6RSH4_STRPU**	*Strongylocentrotus purpuratus*	39	1719
Complement related-long	Purple sea urchin	(33)	
**P69004/ACT2_MESFR**	*Mesocentrotus franciscanus*	164	1611
Actin-15B	Red sea urchin	(115)	
**A0A7M7HL75_STRPU**	*Strongylocentrotus purpuratus*	162	1579
Uncharacterised protein	Purple sea urchin	(117)	
(Actin, cytoskeletal 1A; Actin, cytoskeletal 1B; Beta actin)			
**Q3YL94_MESNU**	*Mesocentrotus nudus*	47	1563
Major yolk protein	Sea urchin	(37)	
**Q964G1_PSEDP**	*Pseudocentrotus depressus*	41	1419
Vitellogenin	Pink sea urchin	(30)	
**A0A7M7GHQ8_STRPU**	*Strongylocentrotus purpuratus*	36	1075
Uncharacterised protein	Purple sea urchin	(30)	
(Tubulin beta chain)			
**A0A7M7PHC8_STRPU**	*Strongylocentrotus purpuratus*	20	766
Uncharacterised protein	Purple sea urchin	(15)	
(Tubulin alpha chain)			
**A0A7M7NNT8_STRPU**	*Strongylocentrotus purpuratus*	27	648
Uncharacterised protein	Purple sea urchin	(19)	
(Histone H4; Histone H2B; Histone H3)			
**A0A7M7THK3_STRPU**	*Strongylocentrotus purpuratus*	14	569
Uncharacterised protein (Heat shock 70 kDa)	Purple sea urchin	(11)	
**Q8MVU0_STRDR**	*Strongylocentrotus droebachiensis*	13	555
Tubulin alpha chain	Green sea urchin	(9)	
**A0A7M6UMT5_STRPU**	*Strongylocentrotus purpuratus*	13	499
Uncharacterised protein	Purple sea urchin	(8)	
(Elongation factor 1-alpha; Translation elongation factor eEF-1 alpha-related centrosphere protein)			
**A0A7M7NZ19_STRPU**	*Strongylocentrotus purpuratus*	13	480
Uncharacterised protein	Purple sea urchin	(10)	
(Glyceraldehyde-3-phosphate dehydrogenase)			
**A0A7M7N257_STRPU**	*Strongylocentrotus purpuratus*	10	452
Uncharacterised protein	Purple sea urchin	(9)	
(Sea urchin Arp3 (SUArp3))			
**A0A7M7T5A0_STRPU**	*Strongylocentrotus purpuratus*	13	429
Uncharacterised protein	Purple sea urchin	(12)	
(Galectin)			
**A0A7M7P855_STRPU**	*Strongylocentrotus purpuratus*	5	276
Uncharacterised protein (Putative 14-3-3 epsilon isoform)	Purple sea urchin	(4)	
**H3IRP0_STRPU**	*Strongylocentrotus purpuratus*	10	261
Histone H2B	Purple sea urchin	(6)	
**A0A7M7PNT6_STRPU**	*Strongylocentrotus purpuratus*	5	235
Uncharacterised protein	Purple sea urchin	(4)	
(Scavenger receptor cysteine-rich protein type 5)			
**Q1PS64_STRPU**	*Strongylocentrotus purpuratus*	4	228
Amassin-2	Purple sea urchin	(4)	
**A0A7M7N0L4_STRPU**	*Strongylocentrotus purpuratus*	6	210
Uncharacterised protein (Villin)	Purple sea urchin	(4)	
**A0A7M6UMU1_STRPU**	*Strongylocentrotus purpuratus*	2	155
Uncharacterised protein	Purple sea urchin	(1)	
(Sodium/potassium ATPase alpha subunit)			
**Q86RA9_STRPU**	*Strongylocentrotus purpuratus*	3	143
Amassin	Purple sea urchin	(2)	
**A0A7M7SSL0_STRPU**	*Strongylocentrotus purpuratus*	3	139
Uncharacterised protein (Heat shock protein gp96)	Purple sea urchin	(2)	
**A0A7M6W5I2_STRPU**	*Strongylocentrotus purpuratus*	3	138
Uncharacterised protein (ATP synthase subunit beta)	Purple sea urchin	(3)	
**A0A7M7NPF8_STRPU**	*Strongylocentrotus purpuratus*	5	130
Uncharacterised protein	Purple sea urchin	(5)	
(C-type lectin domain-containing protein)			
**A0A1D8I2M5_STRPU**	*Strongylocentrotus purpuratus*	3	121
Uncharacterised protein (Catalase-like protein)	Purple sea urchin	(2)	
**A0A7M6UX55_STRPU**	*Strongylocentrotus purpuratus*	3	117
Uncharacterised protein (ATP synthase subunit alpha)	Purple sea urchin	(2)	
**A0A7M6UC86_STRPU**	*Strongylocentrotus purpuratus*	2	116
Uncharacterised protein (Tetraspanin)	Purple sea urchin	(2)	
**A0A7M7NA73_STRPU**	*Strongylocentrotus purpuratus*	2	114
Uncharacterised protein (40S ribosomal protein s27a; Polyubiquitin; Ubiquitin)	Purple sea urchin	(2)	
**A0A7M7NYP9_STRPU**	*Strongylocentrotus purpuratus*	2	112
Uncharacterised protein (L-lactate dehydrogenase)	Purple sea urchin	(2)	
**A0A7M7PPU0_STRPU**	*Strongylocentrotus purpuratus*	2	109
Uncharacterised protein (Actin-related protein 2)	Purple sea urchin	(2)	
**D5H3J3_PSAMI**	*Psammechinus miliaris*	2	102
60S ribosomal protein L40	Green sea urchin/ shore sea urchin	(2)	
**A0A7M7REH8_STRPU**	*Strongylocentrotus purpuratus*	4	98
Uncharacterised protein (Peptidyl-prolyl cis-trans isomerase)	Purple sea urchin	(2)	
**Q5EAJ7_MVP STRPU**	*Strongylocentrotus purpuratus*	2	82
Major vault protein	Purple sea urchin	(2)	
**O06393_STRPU**	*Strongylocentrotus purpuratus*	2	75
Vesicle-fusing ATPase	Purple sea urchin	(1)	
**A0A7M7HNW9_STRPU**	*Strongylocentrotus purpuratus*	1	72
Uncharacterised protein (Amassin-4)	Purple sea urchin	(1)	
**Q26613/EMAP_STRPU**	*Strongylocentrotus purpuratus*	2	70
77 kDa echinoderm microtubule-associate protein	Purple sea urchin	(1)	
**A0A7M7N1D9_STRPU**	*Strongylocentrotus purpuratus*	2	69
Uncharacterised protein (Polyadenylate-binding protein)	Purple sea urchin	(1)	
**A0A7M7LPJ9_STRPU**	*Strongylocentrotus purpuratus*	1	61
Uncharacterised protein (Guanine nucleotide-binding protein G (I) alpha subunit	Purple sea urchin	(1)	
**C4P258_STRPU**	*Strongylocentrotus purpuratus*	2	47
Extracellular transglutaminase	Purple sea urchin	(0)	
A3KLJ5_STRPU	*Strongylocentrotus purpuratus*	1	45
**RNA helicase**	Purple sea urchin	(1)	
B3FNR8_STRPU	*Strongylocentrotus purpuratus*	1	34
**E2E3**	Purple sea urchin	(1)	
**A0A7M7PKS2_STRPU**	*Strongylocentrotus purpuratus*	1	33
Uncharacterised protein	Purple sea urchin	(1)	
(Catalytic subunit of cAMP-dependant histone kinase)			

† Ion score is −10*Log(P), where P is the probability that the observed match is a random event. Individual ion scores >33 indicate identity or extensive similarity (*p* < 0.05). Protein scores are derived from ion scores as a non-probabilistic basis for ranking protein hits.

**Table 2:** Deiminated proteins identified by F95 enrichment and liquid chromatography with tandem mass spectrometry (LC-MS/MS) analysis in coelomic fluid (CF; pool of 3 individual samples) and EVs (pool of 3 individual samples) of purple sea urchin (*Strongylocentrotus purpuratus*); hits are run against the UniProt Echinoidea database. Proteins identified as deimination hits in EVs, coelomic fluid (CF), or both are indicated by a tick (v) for the columns representing EVs and CF, respectively. Uncharacterised hits with an annotated secondary hit are included and indicated in brackets; other unidentified protein hits are not included in this table. Further detailed analysis on number of matches and total score is provided in Supplementary Tables S2 and S3, respectively, as well as full LC-MS/MS results (also including data from a common contaminant database; cRAP 20,190,401; 125 sequences; 41,129 residues) in Supplementary Tables S4 and S5, respectively.

Protein ID	*Species Name*	EVs	CF
Protein Name	Common Name		
**P19615/MYP_STRPU**	*Strongylocentrotus purpuratus*	**v**	**v**
Major yolk protein	Purple sea urchin		
**A0A7M7HL75_STRPU**	*Strongylocentrotus purpuratus*	**v**	**v**
Uncharacterised protein	Purple sea urchin		
(Actin, cytoskeletal 2A; Actin, cytoskeletal 1A; Actin, cytoskeletal 1B; Actin, cytoskeletal 2B)			
**A0A1L3KPZ4_MESNU**	*Mesocentrotus nudus*	**v**	
Beta actin	Sea urchin		
**O18555_HELER**	*Heliocidaris erythrogramma*	**v**	
Cytoplasmic actin CyII	Sea urchin		
**A0A7M6UC80_STRPU**	*Strongylocentrotus purpuratus*		**v**
Uncharacterised protein	Purple sea urchin		
(Histone H2A.V; Histone H2A-bta,sperm)			
**A0A7M7NNT8_STRPU**	*Strongylocentrotus purpuratus*	**v**	**v**
Uncharacterised protein	Purple sea urchin		
(Histone HB2)			
**P07794/H2BL1_PSAMI**	*Psammechinus miliaris*		**v**
Late histone H2B.2.1	Green sea urchin		
**A0A7M7NNT8_STRPU**	*Strongylocentrotus purpuratus*		**v**
Uncharacterised protein	Purple sea urchin		
(Histone H3)			
**H3IPI3_STRPU**	*Strongylocentrotus purpuratus*	**v**	**v**
Uncharacterised protein	Purple sea urchin		
(Histone H4)			
**A0A7M7MZP4_STRPU**	*Strongylocentrotus purpuratus*		**v**
Uncharacterised protein	Purple sea urchin		
(Tubulin alpha chain)			
**A0A7M7GHQ8_STRPU**	*Strongylocentrotus purpuratus*	**v**	**v**
Uncharacterised protein	Purple sea urchin		
(Tubulin beta chain)			
**D5H3J3_PSAMI**	*Psammechinus miliaris*	**v**	**v**
60S ribosomal protein L40	Green sea urchin		
**A0A7M7SSL0_STRPU**	*Strongylocentrotus purpuratus*	**v**	
Uncharacterised protein	Purple sea urchin		
(Heat shock protein gp96)			
**Q7M4J9_HEMPU**	*Hemicentrotus pulcherrimus*	**v**	
98K protein	Sea urchin		
**O443344_STRPU**	*Strongylocentrotus purpuratus*		**v**
Complement C3	Purple sea urchin		
**A0A7M7NVJ2_STRPU**	*Strongylocentrotus purpuratus*		**v**
Uncharacterised protein	Purple sea urchin		
(Fascin)			
**A0A7M6UMT5_STRPU**	*Strongylocentrotus purpuratus*		**v**
Uncharacterised protein	Purple sea urchin		
(Elongation factor alpha-1)			
**A0A1DB8I2L3_STENE**	*Sterechinus neumayery*		**v**
Glyceraldehyde-3-phosphate dehydrogenase	Sea urchin		
**Q26049_PARLI**	*Paracentrotus lividus*		**v**
Cell surface protein	Mediterranean purple sea urchin		

**Table 3:** FoldIndex© analysis of deiminated purple sea urchin proteins identified by F95 enrichment in EVs of sea urchin (*Strongylocentrotus purpuratus*). The number of disordered regions, residue length of the longest disordered region, total number of disordered residues, as well as the number of arginines present in the total number of residues for the individual protein hits are shown. Only protein hits with purple sea urchin are shown in this table; the proteins assessed are highlighted in bold.

Protein Name	Number of Disordered Regions	Longest Disordered Region	Number of Disordered Residues	Number of Arginines
P19615/MYP_STRPU	15	61	290	**63**
**Major yolk protein**				(out of 1357 residues)
A0A7M7HL75_STRPU	3	17	37	**18**
Uncharacterised protein				(out of 376 residues)
(**Actin, cytoskeletal 2A; Actin, cytoskeletal 1A; Actin, cytoskeletal 1B; Actin, cytoskeletal 2B**)				
A0A7M7NNT8_STRPU	1	51	51	**8**
Uncharacterised protein				(out of 122 residues)
(**Histone HB2**)				
H3IPI3_STRPU	1	44	44	**14**
Uncharacterised protein				(out of 103 residues)
(**Histone H4**)				
A0A7M7SSL0_STRPU	9	111	410	**30**
Uncharacterised protein				(out of 806 residues)
(**Heat shock protein gp96**)				
A0A7M7GHQ8_STRPU	4	57	112	**20**
Uncharacterised protein				(out of 447 residues)
(**Tubulin beta chain**)				

**Table 4:** FoldIndex© analysis of deiminated purple sea urchin proteins identified by F95 enrichment in coelomic fluid of sea urchin (*Strongylocentrotus purpuratus*). The number of disordered regions, residue length of the longest disordered region, total number of disordered residues, as well as the number of arginines present in the total number of residues for the individual protein hits are shown. Only protein hits with purple sea urchin are shown in this table; the proteins assessed are highlighted in bold.

Protein Name	Number of Disordered Regions	Longest Disordered Region	Number of Disordered Residues	Number of Arginines
P19615/MYP_STRPU	15	61	290	**63**
**Major yolk protein**				(out of 1357 residues)
A0A7M7NNT8_STRPU	1	66	66	**18**
Uncharacterised protein				(out of 136 residues)
(Histone H4; **Histone H3**; Histone H2B;				
A0A7M7HL75_STRPU	3	17	37	**18**
Uncharacterised protein				(out of 376 residues)
(Actin, **cytoskeletal 2A; Actin, cytoskeletal 1A; Actin, cytoskeletal 1B; Actin, cytoskeletal 2B**)				
O443344_STRPU	15	39	258	**85**
**Complement C3**				(out of 1699 residues)
A0A7M7NRQ3_STRPU	4	57	112	**20**
Uncharacterised protein				(out of 447 residues)
(**Tubulin beta chain**)				
A0A7M7RBS6_STRPU	1	51	51	**8**
Uncharacterised protein				(out of 122 residues)
(**Histone H2B**)				
A0A7M6UC80_STRPU	2	35	41	**12**
Uncharacterised protein				(out of 125 residues)
(Histone **H2A**.V; Histone H2A-bta,sperm)				
A0A7M7MZP4_STRPU	2	49	54	**20**
Uncharacterised protein				(out of 452 residues)
(**Tubulin alpha chain**)				
A0A7M7NVJ2_STRPU	6	51	138	**17**
Uncharacterised protein				(out of 496 residues)
(**Fascin**)				
A0A7M6UMT5_STRPU	2	27	33	**19**
Uncharacterised protein				(out of 461 residues)
(**Elongation factor alpha-1**)				
A0A1DB8I2L3_STENE	0	0	0	**11**
**Glyceraldehyde-3-phosphate dehydrogenase**				(out of 337 residues)

**Table 5:** Top 5 BLASTp results for the predicted PAD protein from the mottled brittle starfish (*Ophionereis fasciata*) genome (all with 100% query cover).

Hit	Protein Accession No.	Species/Family Name	E-Value	Identity (%)
1	WP_111894244	*Arthrospira* sp.	3e-69	99
2	WP_048895331	*Limnospira indica*	4e-69	100
3	CCE20058	*Limniospira indica*	4e-69	100
4	CDM98608	*Limniospira indica*	4e-69	100
5	WP_006622374	*Microcoleaceae*	5e-69	100

## Data Availability

All data supporting the results are included in the paper and the Supplementary Material.

## Supplementary Materials

The following are available online at https://www.mdpi.com/article/10.3390/biology10090866/s1, Figure S1: STRING networks for Cellular GO pathways for: (**A**) Deiminated protein hits in sea urchin EVs; (**B**) Deiminated protein hits in sea urchin coelomic fluid; (**C**) Total protein cargo in sea urchin EVs. Figure S2: Coelomic fluid from sea urchin blotted against human PAD1–6 antibodies. Coelomic fluid (CF) from purple sea urchin was blotted against human PAD1 (ab181762, Abcam), PAD2 (ab50257), PAD3 (ab50246), PAD4 (ab50247), and PAD6 (PA5–72059, Thermo Fisher Scientific UK) antibodies. The clearest reaction was seen using anti-human PAD2 antibody, some reaction was also seen with the PAD4 antibody, while a strong reaction was also seen for PAD6 antibody; protein standard is indicated (std). Table S1: Full LC-MS/MS analysis for total protein cargo analysis from purple sea urchin coelomic fluid EVs. Databases searched were: UniProt database CCP_Echinidea Echinidea_20210511 (38,194 sequences; 24,939,030 residues); an additional search was conducted against a common contaminant database (cRAP 20,190,401; 125 sequences; 41,129 residues). Results are based on data from a pool of 3 individual samples. Table S2: Deiminated proteins in purple sea urchin EVs, identified by F95 enrichment and liquid chromatography with tandem mass spectrometry (LC-MS/MS); information on number of matches and total score are provided. Results are based on data from a pool of 3 individual samples. Table S3: Deiminated proteins in purple sea urchin coelomic fluid, identified by F95 enrichment and liquid chromatography with tandem mass spectrometry (LC-MS/MS); information on number of matches and total score are provided. Results are based on data from a pool of 3 individual samples. Table S4: Full LC-MS/MS analysis for F95-enriched protein hits from purple sea urchin coelomic fluid EVs. Databases searched were: UniProt database CCP_Echinidea Echinidea_20210511 (38,194 sequences; 24,939,030 residues); an additional search was conducted against a common contaminant database (cRAP 20,190,401; 125 sequences; 41,129 residues). Results are based on data from a pool of 3 individual samples. Table S5: Full LC-MS/MS analysis for F95-enriched protein hits from purple sea urchin coelomic fluid. Databases searched were: UniProt database CCP_Echinidea Echinidea_20210511 (38,194 sequences; 24,939,030 residues); an additional search was conducted against a common contaminant database (cRAP 20,190,401; 125 sequences; 41,129 residues). Results are based on data from a pool of 3 individual samples.
