# Supplementary material for: Extracellular Vesicle Signatures and Post-Translational Protein Deimination in Purple Sea Urchin (Strongylocentrotus purpuratus) Coelomic Fluid—Novel Insights into Echinodermata Biology
Source: Biology (Basel). 2021 Sep 3;10(9):866. doi: 10.3390/biology10090866 (PMC8464700; doi:10.3390/biology10090866)
Supplement: Supplementary file 1 [file biology-10-00866-s001.zip › Table S2 EVs F95.pdf]

Supplementary Table S2. Deiminated protein cargo in coelomic fluid EVs of purple sea urchin (*Strongylocentrotus purpuratus*) was identified by F95 enrichment and liquid chromatography with tandem mass spectrometry (LC-MS/MS) analysis. Proteins identified only in EVs and not in coelomic fluid, are highlighted in pink and with an asterisk (*); uncharacterised hits with an annotated secondary hit are included and indicated in brackets; other unidentified protein hits are not included in this table but can be found in Supplementary Table 1. Protein ID, protein name, species hit with the Echinoidea UniProt database, number of matches and total score are included in the table.

**Protein ID**<br>Protein name	***Species name***<br>**Common name**	**Matches (Sequences)**	**Total score (p<0.05)†**
**P19615/MYP_STRPU**<br>Major yolk protein	*Strongylocentrotus purpuratus*<br>Purple sea urchin	9<br>(8)	422
**A0A7M7HL75_STRPU**<br>Uncharacterized protein<br>(Actin, cytoskeletal 2A; Actin, cytoskeletal 1A; Actin, cytoskeletal 1B; Actin, cytoskeletal 2B)	*Strongylocentrotus purpuratus*<br>Purple sea urchin	10<br>(8)	416
**A0A7M7PME7_STRPU**<br>Uncharacterized protein<br>(Major yolk protein)	*Strongylocentrotus purpuratus*<br>Purple sea urchin	8<br>(7)	376
***A0A1L3KPZ4_MESNU**<br>Beta actin	*Mesocentrotus nudus*<br>Sea urchin	9<br>(7)	350
***O18555_HELER**<br>Cytoplasmic actin CyII	*Heliocidaris erythrogramma*<br>Sea urchin	9<br>(7)	328
**A0A7M7NNT8_STRPU**<br>Uncharacterized protein<br>(Histone HB2)	*Strongylocentrotus purpuratus*<br>Purple sea urchin	7<br>(6)	266
**H3IPI3_STRPU**<br>Uncharacterized protein<br>(Histone H4)	*Strongylocentrotus purpuratus*<br>Purple sea urchin	6<br>(4)	184
***A0A7M7SSL0_STRPU**<br>Uncharacterized protein<br>(Heat shock protein gp96)	*Strongylocentrotus purpuratus*<br>Purple sea urchin	2<br>(1)	102
**A0A7M7GHQ8_STRPU**<br>Uncharacterized protein<br>(Tubulin beta chain)	*Strongylocentrotus purpuratus*<br>Purple sea urchin	2<br>(2)	98
**D5H3J3_PSAMI**<br>60S ribosomal protein L40	*Psammechinus miliaris*<br>Green sea urchin	2<br>(1)	69
**A0A7M7RBS6_STRPU**<br>Uncharacterized protein<br>(Histone HB2)	*Strongylocentrotus purpuratus*<br>Purple sea urchin	2<br>(1)	66
***Q7M4J9_HEMPU**<br>98K protein	*Hemicentrotus pulcherrimus*<br>Sea urchin	1<br>(1)	41

† Ions score is – 10*Log(P), where P is the probability that the observed match is a random event. Individual ions scores >33 indicate identity or extensive similarity (p<0.05). Protein scores are derived from ions scores as a non-probabilistic basis for ranking protein hits.
