# Supplementary material for: Extracellular Vesicle Signatures and Post-Translational Protein Deimination in Purple Sea Urchin (Strongylocentrotus purpuratus) Coelomic Fluid—Novel Insights into Echinodermata Biology
Source: Biology (Basel). 2021 Sep 3;10(9):866. doi: 10.3390/biology10090866 (PMC8464700; doi:10.3390/biology10090866)
Supplement: Supplementary file 1 [file biology-10-00866-s001.zip › Table S3 CF F95.pdf]

Supplementary Table S3. Deiminated proteins in coelomic fluid of purple sea urchin (*Strongylocentrotus purpuratus*), identified by F95 enrichment and liquid chromatography with tandem mass spectrometry (LC-MS/MS) analysis, run against Echinoidea database. Proteins identified only in coelomic fluid (but not EVs) are highlighted in pink and with an asterisk (*); uncharacterised hits with an annotated secondary hit are included and indicated in brackets; other unidentified protein hits are not included in this table but can be found in Supplementary Table 2. Protein ID, protein name, species hit with the Echinoidea UniProt database, number of matches and total score are included in the table.

**Protein ID**<br>**Protein name**	***Species name***<br>**Common name**	**Matches**<br>**(Sequences)**	**Total score**<br>**(p<0.05) †**
**P19615/MYP_STRPU**<br>Major yolk protein	*Strongylocentrotus purpuratus*<br>Purple sea urchin	35<br>(28)	1378
**A0A7M7PME7_STRPU**<br>Uncharacterized protein<br>(Major Yolk protein)	*Strongylocentrotus purpuratus*<br>Purple sea urchin	29<br>(23)	1161
**Q7Z1Y6_HEMPU**<br>Major yolk protein	*Hemicentrotus pulcherrimus*<br>Sea urchin	24<br>(20)	906
**A0A7M7NNT8_STRPU**<br>Uncharacterized protein<br>(Histone H4; **Histone H3;** Histone H2B)	*Strongylocentrotus purpuratus*<br>Purple sea urchin	13<br>(11)	414
**A0A7M7HL75_STRPU**<br>Uncharacterized protein<br>(Actin, cytoskeletal 2A; Actin, cytoskeletal 1A; Actin, cytoskeletal 1B; Actin, cytoskeletal 2B)	*Strongylocentrotus purpuratus*<br>Purple sea urchin	11<br>(9)	393
***O443344_STRPU**<br>Complement C3	*Strongylocentrotus purpuratus*<br>Purple sea urchin	7<br>(4)	245
**A0A7M7NRQ3_STRPU**<br>Uncharacterized protein<br>(Tubulin beta chain)	*Strongylocentrotus purpuratus*<br>Purple sea urchin	5<br>(3)	181
***P07794/H2BL1_PSAMI**<br>Late histone H2B.2.1	*Psammechinus miliaris*<br>Green sea urchin	7<br>(6)	161
**A0A7M7RBS6_STRPU**<br>Uncharacterized protein<br>(Histone H2B)	*Strongylocentrotus purpuratus*<br>Purple sea urchin	6<br>(5)	141
**D5H3J3_PSAMI**<br>60S ribosomal protein L40	*Psammechinus miliaris*<br>Green sea urchin	2<br>(1)	105
***A0A7M6UC80_STRPU**<br>Uncharacterized protein<br>(Histone H2A.V; Histone H2A-bta,sperm)	*Strongylocentrotus purpuratus*<br>Purple sea urchin	2<br>(2)	81
***A0A7M7MZP4_STRPU**<br>Uncharacterized protein<br>(Tubulin alpha chain)	*Strongylocentrotus purpuratus*<br>Purple sea urchin	2<br>(1)	81
***A0A7M7NVJ2_STRPU**<br>Uncharacterized protein<br>(Fascin)	*Strongylocentrotus purpuratus*<br>Purple sea urchin	1<br>(1)	68
**A0A7M7HL80_STRPU**<br>Uncharacterized protein<br>(Tubulin alpha chain)	*Strongylocentrotus purpuratus*<br>Purple sea urchin	2<br>(1)	54
***A0A7M6UMT5_STRPU**<br>Uncharacterized protein<br>(Elongation factor alpha-1)	*Strongylocentrotus purpuratus*<br>Purple sea urchin	1<br>(1)	46
***A0A1DB8I2L3_STENE**<br>Glyceraldehyde-3-phosphate dehydrogenase	*Sterechinus neumayery*<br>Sea urchin	2<br>(1)	45
***Q26049_PARLI**<br>Cell surface protein	*Paracentrotus lividus*<br>Mediterranean purple sea urchin	1<br>(0)	33

†Ions score is – 10*Log(P), where P is the probability that the observed match is a random event. Individual ions scores >33 indicate identity or extensive similarity ($p<0.05$). Protein scores are derived from ions scores as a non-probabilistic basis for ranking protein hits.
