# Supplementary figures and images for: Extracellular Vesicle Signatures and Post-Translational Protein Deimination in Purple Sea Urchin (Strongylocentrotus purpuratus) Coelomic Fluid—Novel Insights into Echinodermata Biology
Source: Biology (Basel). 2021 Sep 3;10(9):866. doi: 10.3390/biology10090866 (PMC8464700; doi:10.3390/biology10090866)

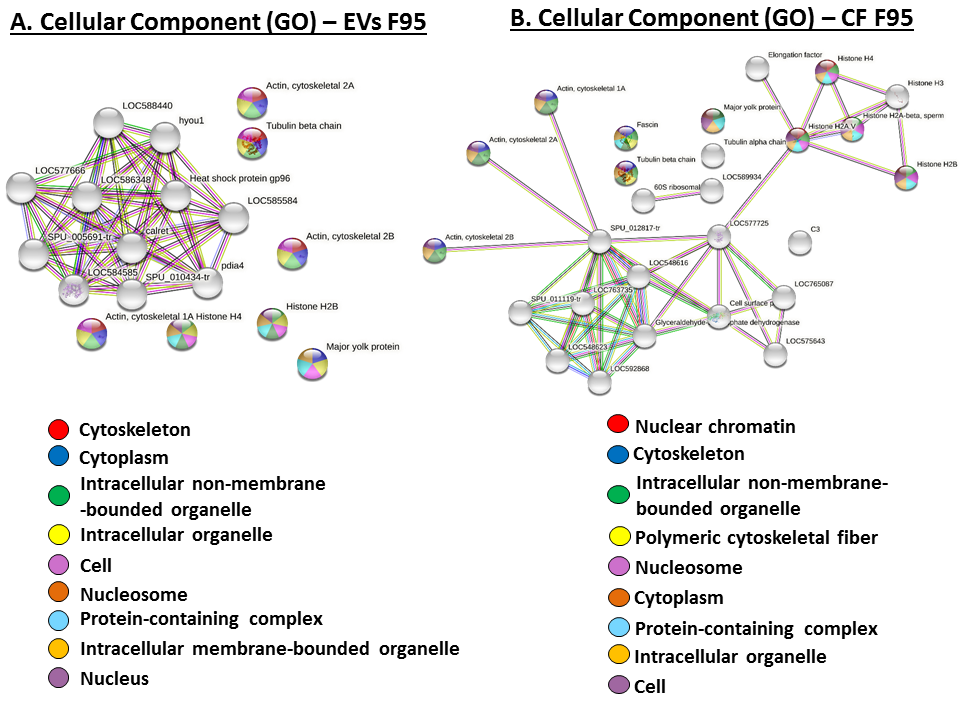

Supplement: Supplementary file 1 [file biology-10-00866-s001.zip › Supplementary Figure S1A-B.tif]

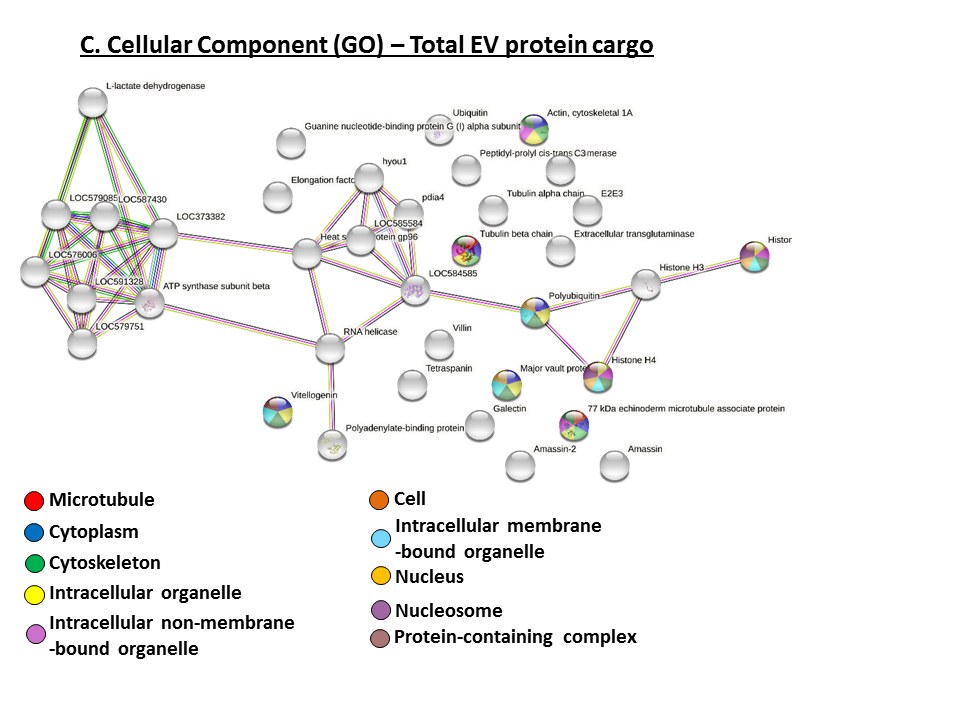

Supplement: Supplementary file 1 [file biology-10-00866-s001.zip › Supplementary Figure S1C.tif]

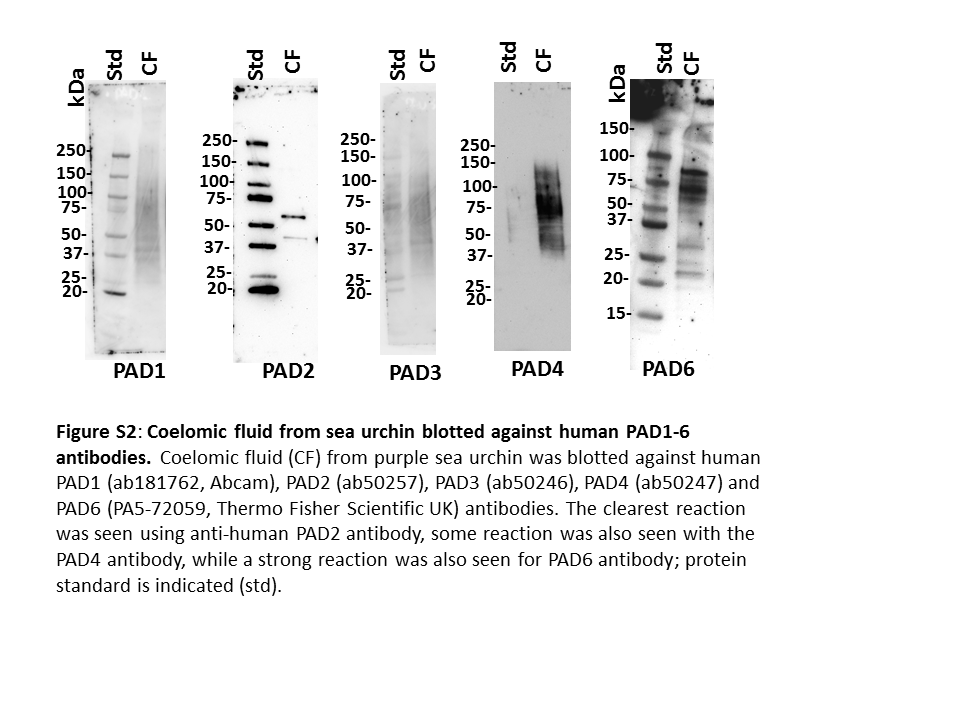

Supplement: Supplementary file 1 [file biology-10-00866-s001.zip › Supplementary Figure S2.tif]
